# Immune correlates of protection for SARS-CoV-2, Ebola and Nipah virus infection

**DOI:** 10.3389/fimmu.2023.1156758

**Published:** 2023-04-17

**Authors:** Beatriz Escudero-Pérez, Philip Lawrence, Javier Castillo-Olivares

**Affiliations:** ^1^WHO Collaborating Centre for Arbovirus and Haemorrhagic Fever Reference and Research, Bernhard Nocht Institute for Tropical Medicine, Hamburg, Germany; ^2^German Center for Infection Research (DZIF), Partner Site Hamburg-Luebeck-Borstel-Reims, Braunschweig, Germany; ^3^CONFLUENCE: Sciences et Humanités (EA 1598), Université Catholique de Lyon (UCLy), Lyon, France; ^4^Laboratory of Viral Zoonotics, University of Cambridge, Cambridge, United Kingdom

**Keywords:** immune correlates of protection, emerging viruses, humoral immunity, cell-mediated immunity, SARS-CoV-2, Nipah virus, Ebola virus

## Abstract

Correlates of protection (CoP) are biological parameters that predict a certain level of protection against an infectious disease. Well-established correlates of protection facilitate the development and licensing of vaccines by assessing protective efficacy without the need to expose clinical trial participants to the infectious agent against which the vaccine aims to protect. Despite the fact that viruses have many features in common, correlates of protection can vary considerably amongst the same virus family and even amongst a same virus depending on the infection phase that is under consideration. Moreover, the complex interplay between the various immune cell populations that interact during infection and the high degree of genetic variation of certain pathogens, renders the identification of immune correlates of protection difficult. Some emerging and re-emerging viruses of high consequence for public health such as SARS-CoV-2, Nipah virus (NiV) and Ebola virus (EBOV) are especially challenging with regards to the identification of CoP since these pathogens have been shown to dysregulate the immune response during infection. Whereas, virus neutralising antibodies and polyfunctional T-cell responses have been shown to correlate with certain levels of protection against SARS-CoV-2, EBOV and NiV, other effector mechanisms of immunity play important roles in shaping the immune response against these pathogens, which in turn might serve as alternative correlates of protection. This review describes the different components of the adaptive and innate immune system that are activated during SARS-CoV-2, EBOV and NiV infections and that may contribute to protection and virus clearance. Overall, we highlight the immune signatures that are associated with protection against these pathogens in humans and could be used as CoP.

## 1 Introduction

The human immune system responds through multiple interactive mechanisms to any invading pathogen, ultimately controlling the virus infection or clearing it from our system and enabling a more rapid response on subsequent encounters with such a pathogen. Correlates of protection (CoP) may be defined as those immunological parameters, characteristic of a specific immune mechanism, that are associated with protection against infection or disease (1). Understanding these specific immune mechanisms can help to identify specific immune CoP, which can then be used as surrogate measurements of vaccine protective efficacy and to assess the susceptibility of individuals and populations to a specific pathogen. It is important to note that whilst protection against different viral infections can be mediated by similar immune effector mechanisms, CoP are specific for a viral disease or infection (or even a specific manifestation of a disease), for a specific population group (elderly vs children) and even for a specific type of vaccine (CoP for an inactivated Influenza A vaccine may not necessarily coincide with that of an intra-nasal live Influenza vaccine, for example) (2–4).

The immune system is classically divided into two branches: the innate and the adaptive immune response. While innate immunity consists of a rapid but less specific inflammatory response, adaptive immunity develops more slowly, but is long-lasting and highly specific (5). However, this is a non-strict dichotomy since both arms of the immune response are strongly inter-connected. The interplay between the different immune cell populations and the complexity of immune reactions renders the rational design of effective vaccines against a specific pathogen difficult. For instance, while protection mediated through antibodies is prominent during the acute phase of an infection, cell‐mediated responses normally play an important role in virus infection clearance and/or during the chronic phase of infection. However, this is not the case for all pathogens (6).

The precise protective role of the different effector mechanisms of the immune system have only been fully characterized for a small number of pathogens. However, this has not prevented the statistical association of specific immune mechanism signatures with protection against a disease manifestation. These statistical correlations are built from data (immunological, virological and clinical readouts), collected from field infections and vaccine clinical trials. Once a statistical association has been made between protection and an immunological biomarker it is difficult to improve, modify or introduce a novel CoP. Therefore, in order to derive reliable CoP, it is necessary first to analyze and review in detail how the immune responses develop in experimental and natural infections. This process would in turn lead to the selection of relevant immunological bio-markers with which to evaluate vaccine efficacy in clinical trials, which ultimately will result in establishing a CoP.

## 2 Types of protective immune responses

There is a wide range of cell populations and soluble factors involved in the development of a protective immune reponse against a particular pathogen. Each of these immunological parametres is measurable and constitutes the basis from which to define a CoP. Hereinafter, we describe the main players of the immune responses that can lead to pathogen clearance and protection against disease.

### 2.1 Innate immunity

Innate immunity is the first line of defense against invading pathogens. There are several cell types involved in the innate immune response: monocytes, dendritic cells (DC), macrophages, mast cells, basophils, eosinophils, natural killer (NK) cells and innate lymphoid effector cells. Other than the anatomic and physiologic barriers, innate immune responses comprise endocytic or phagocytic and inflammatory processes as defense mechanisms (7). Both phagocytic and inflammatory immune processes promote the clearance of pathogens and activation of the adaptive immune response (7–9).

For instance, the innate immune response may lead to the activation of the complement cascade which will induce the opsonization of certain pathogens thus rendering them susceptible to phagocytosis, enzymatic degradation or lysis in the case of bacterial pathogens. Another mechanism used during the innate immune response is the production of cytokines and chemokines. This process can lead to an inflammatory state with local activation of cellular responses and recruitment of additional immune cells to sites of infection. The presence of foreign nucleic acid molecules, such as double-stranded RNA (dsRNA) inside the cell, precedes the secretion of interferons (IFNs), the major soluble factors of the innate immune response.

There are several types of IFN but the most notable in virus clearance are type I IFN, which includes IFN-α and IFN-β, IFN type II (IFN-γ) and IFN type III (IFN-λ) (10). The main role of IFN consists in inhibiting viral replication in cells that are already infected but it can also contribute to the protection of neighboring, uninfected cells. Interferon activates signaling pathways that lead to the degradation of the invading pathogen and the activation of some kinase proteins that will shut down the embattled host cell, thus inhibiting viral replication without killing the cell. In some cases, however, the infected cell can also die to prevent viral replication. Hematopoietic cells are the main producers of IFN-α and amongst them, plasmacytoid DCs (pDCs) are the major source (11). On the other hand, most infected cells are capable of producing IFN-β (12). However, the function of innate immune cell populations can be significantly affected during certain infections.

Early innate immune responses do not represent an isolated compartment of the immune system since their activation influences the type of adaptive immune response that develops during the course of the infection (9).

Thus, since soluble factors, molecules and cells involved in the innate immune response influence the development of specific effector mechanisms of the adaptive immune response (antibodies, T cells, immunological memory), which typically define CoP, the measurement of specific innate immune response signatures could potentially be used during an early phase of a vaccination trial to determine early on the protective capacity of a specific vaccine. In other words, innate immune response signatures can serve as alternative CoP. This is an area that has not been sufficiently exploited so far and deserves further investigation.

#### 2.1.1 Innate effector cells

Innate effector T cells can act without previous exposure to antigens. Examples of innate effector T cells include invariant natural killer T (iNKT) cells and γδ T cell receptor expressing cells. Despite their limited T cell receptor (TCR) diversity and low capacity for proliferation, these cells can rapidly execute effector functions, such as the release of various cytokines, chemokines and growth factors. These mechanisms can initially control infection, interact with the adaptative immune response and even promote the development of effector and memory T cells (13). An example of a robust γδ T cell and effector memory T cell response is that observed after smallpox vaccination (14).

### 2.2 Adaptive immunity

The adaptive immune system, by virtue of one of its most defining features, immunological memory, enables a fast and effective response against an invading pathogen upon a second exposure to that pathogen and, in many cases, confers long lasting protection. However, for some diseases, it has been shown that protection declines over time. Whilst the level of circulating antibodies is commonly used as a CoP to assess the protective efficacy against many viral diseases, there are instances where protection has been observed in the face of very low virus-specific antibody titers. In these instances, measurements of virus-specific memory B-cell frequency might serve as more realistic CoP than simply measuring serum antibody levels. In other cases, cellular immunity might play a more important protective role than previously recognized. Indeed, during Influenza virus infections it has been clearly demonstrated that virus-specific T-cell responses limit the severity of disease (15).

As a general rule, antibodies tend to prevent cell infection whereas cellular immune responses rather act once replication of the pathogen takes place (16, 17).

#### 2.2.1 Humoral immunity

Humoral immunity results in the production of antibodies that target specific pathogens. There are 5 isotypes or classes of antibodies, IgA, IgD, IgE, IgG and IgM, that have different biological functions. This classification is made according to their heavy chain, namely alpha, delta, epsilon, gamma or mu respectively (7). Additionally, antibodies can be subdivided into several subclasses (IgG1, IgG2, IgG3, IgG4, IgA1, IgA2) that are structurally and functionally different. While the Fab region of an antibody performs mostly a recognition and/or neutralization role, the Fc region is rather used in cell-mediated immune functions (18, 19).

After a first encounter with a pathogen, B cells will differentiate into effector B cells or plasma cells, which will then secrete antibodies specific to the pathogen encountered. A fraction of these cells will then become memory B cells which are long-lived and can respond quickly after a second exposure to the pathogen. IgG antibody production against a first time encountered pathogen can take up to two weeks to develop, however, if re-infection occurs, antibodies are produced after only a day or two thanks to these antigen-specific memory cells (20).

Antibody production is generated by B lymphocytes, however, CD4+ T cells are required in this process. When antigen specific CD4+ helper T cells interact with activated B cells, they produce IL-4 and IL-5 that will then induce B cell proliferation and antibody production. These antibodies can bind to pathogens and prevent their proliferation through different mechanisms such as neutralization, opsonization and complement activation. Neutralization consists of the binding of antibodies to the surface of the pathogen, thus blocking the pathogen’s attachment to the cell or interfering with virus uncoating within the cell. Opsonization on the other hand, requires the pathogen to be ‘marked’ by opsonins (such as IgG antibody, C3b or C1q molecules of the complement) for the subsequent phagocytic removal of the pathogen (21). Complement-dependent cytotoxicity (CDC) takes place when complement protein C1q (in the classical complement pathway), C3b (in the alternative complement pathway) or Mannose binding lectin (MBL), in the Lectin complement pathway, bind to the Fc region of IgG or IgM, coupled to a pathogen antigen expressed on the surface of an infected cell. This activates the complement pathway that will lead to the formation of a membrane attack complex (MAC) that will then cause cell lysis (22, 23).

In some cases, B cells can direct other immune cells to eliminate the pathogen *via* Fc-Fc receptor (FcR) interactions, thus combining the strong antiviral functions of innate immune effector cells with the specificity of the adaptive humoral activity. These mechanisms comprise Antibody Dependent Cellular Cytotoxicity (ADCC) and Antibody Dependent Cellular Phagocytosis (ADCP) (24). During these cell-mediated immune mechanisms, antibodies are produced that will bind the pathogen and these will then be recognized by effector cells that have FcR, namely NK cells, neutrophils, macrophages and dendritic cells. In the case of ADCC, the effector cell will lyse the targeted cell containing the pathogen on the surface coated with IgG1 or IgG3 containing the bound Fc. Pathogen infected cells can be eliminated through the action of cytokines, reactive oxygen species (ROS), perforin and/or granzymes. In contrast, during ADCP, the targeted cell will be engulfed and processed for phagolysosomal degradation. The main leukocytes involved in ADCP include monocytes, macrophages, neutrophils, and eosinophils (25, 26). In addition, B cells can activate and present antigens directly to effector T cells. B cells can directly recognize certain antigens *via* their surface IgG. These specifically bound antigens will be endocytosed, processed and their peptides presented to specific antigen matching T helper cells. As a result of this interaction, B cells express costimulatory molecules that can activate the T helper cells that will then coordinate effector functions.

It is also important to mention the role of immune memory, which during certain infections can be highly correlated with protection. B cell memory is generated by two different cell subsets: memory B cells and long-lived plasma cells or memory plasma cells. Thus, upon a second antigen exposure, memory plasma-cells can rapidly produce antibodies and memory B cells can differentiate faster into plasma cells and start a quick and robust response producing antibodies, isotype switching, effector functions and affinity maturation besides rapid proliferation (27). These processes can play an important role when low, pre-existing antibody levels are present or if the existing antibodies are overcome by the infectious agent (8, 16).

The ability to induce a strong humoral response is the hallmark of an effective host defense against certain infections (7). There are several factors that may affect the efficiency of the antibody response such as the titer, location, subclass of antibody, time of appearance and durability. However, the specific threshold levels of antibody titers conferring protection against many specific pathogens are either currently undetermined or variable amongst pathogens (28). Nevertheless, due to the ease of measuring antigen-specific antibody levels in various clinical specimens and bodily fluids, CoP based on antibody level measurement (e.g. virus neutralization, antibody binding assays, hemagglutination inhibition assays) have been used extensively to assess the immunity of populations against a specific pathogen and to evaluate vaccine efficacy. Furthermore, collection and processing of clinical material for antibody analysis is relatively simple in comparison to collecting, storing and processing PBMC for the assessment of cell-mediated immune responses.

#### 2.2.2 Cell-mediated immunity

Most infectious pathogens are susceptible to the action of antibodies during the extracellular phase of their infection cycle. However, humoral immune responses are not completely effective at clearing pathogens when they are inside cells and cell-mediated immune effector mechanisms are called upon to clear viral infection. These mechanisms are mediated typically by CD8+ cytotoxic T-lymphocytes, which bind in a specific manner *via* their T-cell receptors, to the MHC-I molecules of infected cells that display viral antigen-derived peptides. However, this is not the only cell-mediated effector mechanism of T lymphocytes. Indeed, upon encountering infected cells, T cells secrete pro-inflammatory cytokines, co-stimulatory soluble factors and other regulatory signals. Thus, cell-mediated immunity (CMI) is relevant for intracellular pathogens and this protective mechanism can also synergize with an antibody production strategy in order to achieve a protective response. For instance, it has been shown that certain antibodies can activate Th1 cells through FcR, thus facilitating the rapid processing of antigens (29). Likewise, as alluded to earlier, ADCC and ADCP can also be considered as hybrid effector mechanisms of immunity involving the synergistic action of antibodies and innate immune effector cells.

T cells are considered as the main mediators of cellular adaptive immune responses. They have a crucial role in immunosurveillance since they can discriminate pathogen-derived peptides from native “self” proteins. In order to mount an efficient immune response, after recognition of foreign peptides these cells undergo activation.

During infection, antigen-presenting cells (APCs) recognize and process invading pathogens thus presenting these foreign epitopes to T cells through major histocompatibility complex (MHC) molecules. Such peptides can only be recognized by T cells when they are presented by MHC molecules. There are two types of MHC: class I, which is expressed on the surface of all nucleated cells, and class II which is located on surfaces of specialized APCs. CD8+ and CD4+ T cells will bind MHC I and MHC II respectively.

**Figure 1:**
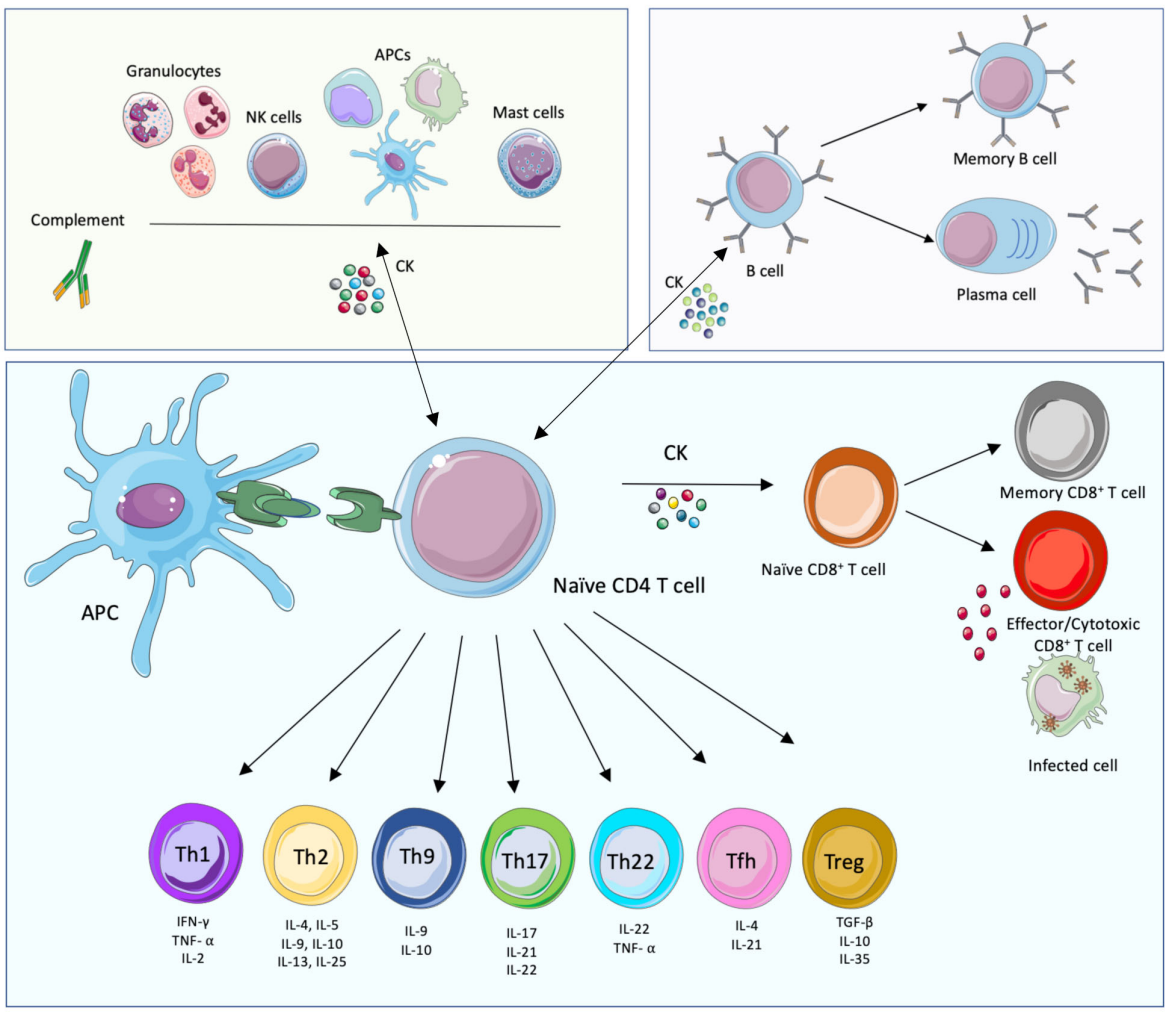
Innate, humoral and cellular-mediated immune responses. The main cellular immune players of the cell-mediated innate (green background, top left), humoral (purple, top right) and adaptative cell-mediated (blue, bottom) immune responses and their interconnections are displayed. The components of the innate immune system provide, together with their effector functions and soluble mediators, an immediate response to pathogens. This response triggers in turn the adaptive immune system, mostly T cell-mediated immune responses that lead to the activation of effector T cells and the activation of B cell functions. This branch of immunity provides specific, long-lasting immune responses. The adaptive and innate immune systems are connected; importantly, while soluble mediators are important to link both arms of immunity, the presentation of foreign peptides (in green) by Antigen Presenting Cells (APCs) is also necessary, together with immune mediators such as cytokines (CK). This figure was created with smart.servier.com.

CD4+ T cells play a central role in the development of the adaptative immune response since they direct downstream effector mechanisms of other immune cells through the secretion of different types of cytokines and chemokines. Through their MHC-II molecules, APCs can present pathogen peptides to naïve CD4+ T cells. If activated, APCs then provide specific co‐stimulatory signals resulting in T cell proliferation and differentiation of naïve CD4+ T cells into specific functional T helper (Th) cell subsets, namely: Th1, Th2, Th9, Th17, Th22, T follicular helper (Tfh) and regulatory T (Treg) cells, amongst others (**Figure 1**). These Th cells contribute to immunoregulation of inflammatory, humoral or CMI responses through the release of effector molecules. A Th1 response for instance, involves the release of tumor necrosis factor-alpha (TNF-α), IFN-γ, and interleukin-2 (IL- 2) amongst others, that will mainly help to clear intracellular pathogens. Th2 responses will release IL-4, IL-5, IL-6 and IL-13, that are mainly involved in the clearance of extracellular pathogens. Th17 effector cells will secrete IL-17, IL-21 and IL-22 and are responsible for the clearance of some extracellular pathogens, however they are also involved in auto immune processes. On the other hand, Treg cells are involved in tolerance and secrete mainly TGF-β and IL-10. It is important to take into consideration that the release of the mentioned cytokines is not exclusive to T cells and that some other immune cells are also an important source of cytokines and chemokines that play crucial roles during infection. In addition, CD4+ T cells also release soluble factors that contribute to the generation and maintenance of CD8+ T cells (30).

As mentioned earlier, cytotoxic CD8+ T lymphocytes (CTL) also play an important role during CMI responses. When activated through MHC I presentation of certain intracellular antigens, these cells release cytotoxic proteins such as perforin, granzyme and cytokines including IFN-γ, TNF-α, IL-2, IL-4, and IL-10, that trigger the killing of specific target cells (8, 31).

Once infection is cleared, antigen‐specific effector T cell (CD4+ and CD8+ T) populations decline and a small cellular subset is maintained as antigen‐specific effector and long-lived memory T cells (CD4+ and CD8+ T cells) (32). Hence, in a secondary immune response the numbers and the activation status of T cells rapidly increase, the stimulatory antigen requirement to induce a response is reduced and as a consequence, a faster response of the effector functions takes place compared to a first contact with the pathogen (32). There are two main subpopulations of memory cells: effector-memory T cells and central-memory T cells. Effector memory T cells circulate through non-lymphoid tissues and provide an immediate response at pathogen sites of entry, but they have a poor proliferative capacity. Central memory T cells, on the other hand, are located in secondary lymphoid tissues, have a long-life span and a high proliferative capacity. Together, effector and central memory T cells have been shown to protect and reduce infection levels in several vaccine studies (32, 33).

Factors such as T-cell phenotype, which antigen the cells are specific to and their function can influence the potential of the CMI response to be an immune CoP during infection. Thus, T cell proliferation and the specific cytokine profile secreted by immune cells in response to specific antigens could be used as CoP for certain infections.

## 3 Protective immune responses during SARS-CoV-2, Nipah virus and Ebola virus infection

### 3.1 SARS-CoV-2

Severe Acute Respiratory Syndrome Coronavirus 2 (SARS-CoV-2) is an enveloped virus with a positive single-stranded RNA genome that belongs to the *Coronaviridae* family and the ß-Coronavirus genus (34).

SARS-CoV-2 is transmitted mainly *via* respiratory droplets and can cause the syndrome known as coronavirus disease 2019 (COVID-19). While most patients are asymptomatic or mildly symptomatic with flu-like symptoms, 13.9% of patients can experience complications that may lead to acute respiratory distress syndrome (ARDS), disseminated intravascular coagulation (DIC) or organ-failure, amongst others (35, 36). This, together with its high transmissibility, makes this virus a public health threat for humans.

Upon entry into a host’s cells, SARS-CoV-2 causes cell damage and triggers a host immune response. There are several molecular mechanisms by which the human immune system can be hijacked by SARS-CoV-2. In this way, innate immune responses are affected, adaptive immune responses are delayed and as a consequence, viral clearance is inefficient and the virus can spread systemically.

#### 3.1.1 The role of cytokines in SARS-CoV-2 infection

While inflammation is crucial for the development of an efficient and coordinated antiviral immune response, an exacerbated inflammatory response can become detrimental for the host. This is the case for SARS-CoV-2 infection where a ‘cytokine storm’ signature has been shown to be a common denominator in severe cases of COVID-19 (37–40). These high levels of proinflammatory cytokines have been associated with injury and loss of lung function, increased levels of SARS-CoV-2 load and severe or fatal outcomes (41). Some proinflammatory markers that are elevated during severe COVID-19 include IL-6, IL-8, IL-1β, TNF-α, MCP-3, TGF-β, CXCL10 and IL-17, amongst others (42, 43). Severe cases of SARS-CoV-2 also correlate with the release of ROS. It is believed that ROS, in turn, increases the expression of proinflammatory cytokines, which further contribute to disease severity (44, 45). The elevated expression of multiple chemokines during COVID-19 also leads to high numbers of neutrophils and monocytes, which in severe cases, can infiltrate the alveolar spaces and are believed to contribute to lung injury and increased disease severity (46, 47). Moreover, this cytokine storm also has an impact on the adaptive immune response, since the low expression of HLA-DR induced by high concentrations of IL-6 and TNF-α leads to a pronounced lymphopenia in severe COVID-19 cases (48).

Amongst cytokines, it is also important to mention IFN. Despite its importance in viral clearance, the precise role of IFN during COVID-19 has still not been elucidated; IFN-I production can be partially impaired by SARS-CoV-2 proteins such as the M protein or non-structural proteins nsp1, nsp6, nsp13-15 and orf6 (49–51). The efficacy of IFN during COVID-19 is however controversial, while some studies have shown a potential protective role during SARS-CoV-2 infection (52, 53), others have suggested that it may be detrimental during infection (54). For instance, it has been shown that high levels of IFN-α are associated with high viral loads and severity, thus indicating that in some severe cases, IFN signatures fail to clear the viral load (55). However, this could be explained by a delayed production of IFN when SARS-CoV-2 titers are already too high and thus IFN cannot clear the virus.

On the contrary, it seems that an early production of IFN and an efficient adaptive immune response correlate with control of SARS-CoV-2 infection, whilst both absence or prolonged presence of IFN can lead to cellular hyperactivation with high inflammation levels that may cause a detrimental clinical outcome (56). Some other studies have suggested a link between severe human cases of COVID-19 and defective IFN responses (52, 57, 58). For instance, it has shown that the impairment of IFN responses, caused by insufficient production of IFN or the presence of autoantibodies against interferons in the host, is correlated with COVID-19 severity (58, 59). In agreement with an IFN protective role, several treatments have been shown to alleviate the severity of COVID-19 by accelerating viral clearance and decreasing levels of certain pro-inflammatory cytokines (60–63). Interestingly, a recent study showed that treatment with a single subcutaneous injection of pegylated interferon lambda in (majoritarily vaccinated) patients with acute COVID-19 decreased disease severity by 51% when compared with placebo-treated patients (64). While not all aspects of IFN effects during COVID-19 are clear, it would appear that IFN treatment during COVID-19 is more efficient in the early phases of the disease (65–67).

In addition to the timing of IFN production or its presence or absence during COVID-19, the anatomical location of IFN also seems to be relevant to determine disease severity. Currently, complete data regarding the circulating levels of IFN-I in association with the severity of COVID-19 disease are lacking. Some studies have shown that circulating IFN-α levels were not significantly different between severe and mild cases when measured in plasma (68) and that prolonged IFN production during SARS-CoV-2 infection can be detrimental for the host and cause negative clinical outcomes (54, 69). However, with regards to local IFN lung production, high IFN-III levels in the upper respiratory tract have been seen to be protective during COVID-19 resulting in mild cases, while high IFN-I and IFN-II levels in the lower respiratory tract have been associated with severe cases of COVID-19 (70). Other factors, such as the age of the patients, seem to be relevant for the severity of the disease. This could be related to the fact that IFN production is impaired by age through the decrease in RIG-I signaling efficiency and pDC IFN production capacity (71). For instance, in a recent study on SARS-CoV-2 infected macaques, it was shown that, in aged macaques, there was higher expression of pro-inflammatory cytokines, lower IFN-I and increased lung pathology (72).

Therefore, the role of IFN during COVID-19 may be strongly influenced, in addition to its presence or absence, by its anatomical location, the moment at which it is produced and the pre-existing cytokine host environment and pre-immune status. Thus, depending on the host and disease context, IFN kinetics can result in a protective or detrimental outcome.

#### 3.1.2 The role of T cell immunity in SARS-CoV-2 infection

Adaptive immune responses are essential in controling and clearing SARS-CoV-2 infection, thus cellular and humoral immunity can confer protection during COVID-19. However, adaptative immune responses are highly influenced during infection by multiple factors, including the immune status of the host (genetic and acquired factors), the efficacy of the innate immune response and the initial virus load, amongst others (73–75).

T cell responses seem to correlate with protection during SARS-CoV-2 infection, however, they are partially impaired in severe cases of COVID-19 thus leading to exacerbated activation and lymphopenia (56). Although the mechanisms responsible for this phenomenon are not fully understood; it would appear that lymphocyte hyperactivation, exhaustion and impaired lymphocyte proliferation can contribute to disease, especially in severe COVID-19 cases (76, 77). The type of T cell response seems to also be relevant. While a biased Th1 phenotype seems to be associated with milder COVID-19 cases and good clinical outcome, Th2 and Th17 responses have been shown to be more prominent and detrimental in severe cases (76, 78). However, it is difficult to draw a general conclusion from such studies, as the observations are often based on relatively low numbers of patients.

In terms of cellular immunity, it is important to identify SARS-CoV-2 specific epitopes that elicit efficient responses in humans. The immunodominance and immunoprevalence of a peptide correspond respectively to how strongly and how frequently a given peptide sequence is recognized by T cells. In the context of SARS-CoV-2, it is possible that an optimized immunodominance and immunoprevalence could improve the efficiency of host immune responses. Therefore, by knowing which specific epitopes can elicit an efficient T cell response, it is possible to modulate the immune responses, thus possibly improving the outcome of the disease by providing immunological memory.

It has previously been shown that convalescent COVID-19 patients harbor an efficient CD4+ T cell response against the SARS-CoV-2 spike (S) glycoprotein. This response also seems to correlate with the presence of specific IgG and IgA titers. Interestingly, several studies showed that some individuals unexposed to SARS-CoV-2 had S specific CD4+ T cells and, at a low level, specific CD8+ T cells (79–81). The presence of these specific responses in non-previously infected individuals could be explained by cross-reactivity responses from previous coronavirus infection. In support of this, an additional study showed that some human samples obtained before the SARS-CoV-2 virus pandemic harbor preexisting memory CD4+ T cells that are cross-reactive to specific SARS-CoV-2 epitopes but also to other common cold coronaviruses (82). Of note, while CD4+ T cells from healthy donors mostly target the C terminal part of the S glycoprotein, CD4+ T cells from COVID-19 patients target almost equally both the N- and C-terminal parts of the SARS-CoV-2 S glycoprotein. This is probably due to the fact that the C-terminal part of the S glycoprotein of many betacoronaviruses has high homology (83). Another difference is that CD4+ T cells from convalescent patients from mild to severe COVID-19 are in an activated state (81). In addition, S glycoprotein-specific T CD4+ cell responses are considered to support antibody generation, thus correlating cellular with humoral immunity in the memory phase (84). Whether S specific T cell responses provide a potential protective role or modulate the severity of the disease in healthy individuals when exposed to SARS-CoV-2 remains to be determined. Another mechanism of protective immunity that has been asasociated with protection against COVID-19 is the presence of resident memory T cells in the lungs, which can last up to 10 months post-infection regardless of the severity of COVID-19 (85, 86).

CD8+ T cells responses seem to be highly heterogeneous between COVID-19 patients. A correlation has been shown between a high expression level of effector molecules by CD8^+^ T cells and a positive clinical outcome (78). This is supported by non-human primate (NHP) models, where, in SARS-CoV-2 infection in macaques, CD8^+^ T cell responses have an important role in protection even when neutralizing antibody levels are low (87). The relevance of CD8+ T cells was also supported in recovered COVID-19 patients, where they were shown to harbor not only SARS-CoV-2-specific CD8^+^ T cells, but also CD8^+^ T cell memory cells (88, 89). Likewise, clonal expansion of CD8+ T cells has been suggested to be present in mild COVID-19 cases (56). Several studies have identified the importance of respiratory CD8^+^ T cell responses and the importance of the interaction between cytotoxic CD8+ T cell and epithelial cells in the upper respiratory tract (90).

In conclusion, severe COVID-19 cases correlate with a delayed and excessive adaptive immune response, whilst in milder and convalescent cases, it appears of importance to have an early robust T cell response that leads to SARS-2 clearance. Moreover, T cell responses are probably not redundant thus cellular and humoral responses can be simultaneously considered as CoP.

#### 3.1.3 The role of B cells in SARS-CoV-2 infection

In general, a specific level of circulating anti-viral antibodies is necessary to confer humoral protection against infection. Most patients with COVID-19 develop IgM and IgG within days to weeks after the onset of symptoms (91). However, the relevance of SARS-CoV-2 specific antibodies during infection is not yet clear.

The protective effect of humoral immune responses against SARS-CoV-2 re-infection depends on how long the humoral response lasts and the antigenic characteristics of the re-infecting virus. After natural, SARS-CoV-2 infection, virus-specific T cells, memory B cells and protective neutralizing antibodies can be detected more than 1 year after infection (92–94). In general, memory B cells and specific protective antibodies are still present at 12-18 months post-infection (at least as long as the studies lasted). Indeed, in one study, 20 months after initial SARS-CoV-2 infection, natural antibody and cellular immunity were still shown to confer protection against infection and hospitalization in 95% and 87% of cases respectively compared to patients that presented no immunity (95). In comparison, vaccine-induced immunity decays faster than natural immunity. Thus, after vaccination a hook effect is observed, while protection is very efficient in the first months, it has been shown to decline more rapidly, nearly disappearing five months after the second dose (96, 97). Moreover, after vaccination the immunogenic reaction takes place against the spike S protein only and IgA is minimally elicited.

With regards to the natural humoral response, it was shown that 300 days after natural infection, IgG antibodies against SARS-CoV-2 S and N proteins were present in 68% and 87% of subjects respectively (98). In fact, many studies have shown the presence of SARS-CoV-2 IgG neutralizing antibodies months after contracting COVID-19 (88, 99–103). Importantly, recent studies have detected the presence of neutralizing IgAs on the surface of the upper nasopharyngeal airway mucosa, lasting for several months (104, 105).

When hybrid immunity takes place (natural + vaccination immunity) the data is slightly contradictory. Some studies show that vaccination in recovered COVID-19 patients improves the disease outcome or increases antibody titers (106–110). For instance, in the previous mentioned (95) study, hybrid immunity induced by either one or two doses of a COVID-19 vaccine was associated with an additional risk reduction of SARS-CoV-2 reinfection compared with natural immunity for up to 9 months, although with small absolute differences. An additional study has shown that infection probability after vaccination is significantly lower than the possibility of reinfection after natural infection (111). For instance, it has also been shown that hybrid immunity is 95.3% and 97.4% effective in preventing hospital admission and severe disease respectively at 6 and 12 months, with the first vaccination dose after the most recent infection. With regards to reinfection, hybrid immunity effectiveness after primary vaccination decreased to 46.5% and 41.8% at 6 and 12 months respectively (112). On the contrary, several studies have shown no statistically significant differences between natural or hybrid immunity effectiveness in terms of increase in neutralizing antibodies, cellular immunity, or specific memory B cells in recovered COVID-19 patients after the second vaccine dose (113–116).

Age is also an important factor with regards to antibody titers against SARS-CoV-2. It has been shown that adaptive immune humoral responses wane with age not only after COVID-19 illness but also after vaccination (117–119).

Overall, there are many studies determining the potential protective role conferred by previous infection and/or vaccination and while there is conflicting evidence, it has been shown that in most cases the presence of high antibody titers decreases the risk of infection by SARS-CoV-2, albeit this risk is not completely eliminated. These studies are summarized in (120). Importantly, when reinfection takes place, previously SARS-CoV-2-exposed patients or recently vaccinated individuals seem to be protected from relevant clinical repercussions and against infection by certain variants (121–123). In these studies, neutralizing antibody titers correlated with the level of protection and thus this parameter is often used as a CoP for COVID-19. In contrast, with regards to the potential protection of SARS-CoV-2 pre-existing humoral immunity towards new molecular variants of the virus, some studies in rhesus macaques have shown that neutralizing antibodies developed during a first SARS-CoV2 infection confer clinical protection against some of the new variants (124–126). Moreover, there are indications that existing humoral cross-reactivity does occur between SARS-CoV-2 and other Beta-coronaviruses. For instance, it has been shown that neutralizing antibodies from the 2003 SARS-1 outbreak can neutralize SARS-CoV-2 (82).

It is also important to consider that one-fifth of SARS-CoV-2 infections result in long-term COVID-19, where despite viral clearance, certain symptoms persist and can lead to post-acute sequelae of COVID-19 (PASC). The dysregulation of the host immune response and virus persistence are believed to account for the development of PASC. Among the immune responses noted in PASC, a distinct humoral immune response was observed, with more avid IgM, weaker Fcγ receptor binding anti-SARS-CoV-2 antibodies and an expanded inflammatory antibody response recognizing the human Betacoronavirus OC43 that can cross-react across SARS-CoV-2 and other coronaviruses. In some cases, CD8+ T cells against Cytomegalovirus (CMV) and Epstein-Barr virus (EBV) reactivation have also been detected (127, 128). The mechanism by which these markers lead to PASC are still not well known and may involve different pathophysiological mechanisms that translate into PASC being an heterogenous syndrome with specific endotypes.

In summary, protection against SARS-CoV-2 depends on a coordinated immune response involving various effector mechanisms of the adaptive immune system. The information acquired to date on SARS-CoV-2 immunity is enabling the development of effective treatments and vaccines to reverse the detrimental immune responses sometimes associated with infection. In conclusion, protection against SARS-CoV-2 depends on: (i) eliciting an early non-exacerbated, innate immune response with limited early IFN production, (ii) inducing a robust cellular response without hyperactivation of T cells, (iii) inducing an effective humoral response with neutralizing antibody production, (iv) generating immunological memory and (v) producing cross-reactive, non-specific innate and adaptive immune responses to generate heterologous protection against COVID-19. The main COVID-19 immune CoP candidates are displayed in **Supplementary Table 1**.

#### 3.1.4 SARS-CoV-2 variants and their interplay with immune responses

The increasing SARS-CoV-2 genomic diversity poses a potential threat to vaccination efficiency since antigenic changes can lead to the appearance of variants of concern (VOCs) with improved viral fitness that can jeopardize a host’s immunity in comparison to previous circulating strains. VOCs sometimes have significant mutations that give them unique properties with a functional impact affecting virus-host interactions and infection capacity, transmission and/or replication, amongst others. As of March 2023, the following major VOCs have been indentified: Alpha (B.1.1.7), Beta (B.1.315), Gamma (P.1), Delta (B.1.617.2), and Omicron (B.1.1.529. */BA.*) (129).

Interestingly, SARS-CoV-2 variants have been shown to differ in their capacity to bind to the SARS-CoV-2 receptor Angiotensin-converting enzyme 2 (ACE 2), in their antibody escape capacity or in triggering different host immune responses. For instance, while the first SARS-CoV-2 variants tended to induce a stronger innate immune response, SARS-CoV-2 Delta has integrated multiple improved mechanisms to evade an IFN response by suppressing the host innate immune response (130).

SARS-CoV-2 Omicron has recently been shown to include many concerning mutations that affect several viral proteins. SARS-CoV-2 proteins can be classified into three categories: structural, non-structural and accessory proteins. Structural SARS-CoV-2 proteins notably play a role in virion assembly and formation. There are four major SARS-CoV-2 structural proteins: the spike protein S, the envelope protein E, the membrane protein M and the nucleocapsid protein N. The Omicron variant has been shown to contain unique mutations mainly in the receptor-binding domain (RBD) and the N-terminal domain of the S1 spike subunit (131). Omicron spike mutations increase binding to ACE 2 and enable antibody escape, thus adding an increased immune evasion capacity to an already higher transmission and replication fitness. While the most explored mutations are found in the spike protein, further mutations in Omicron have also been detected in the N-terminus region of the structural E protein which is known to interact with NSP3 for ubiquitination and glycosylation (132), and also in the M protein that promotes the assembly of new viral particles, affecting membrane integrity and post-translational modifications (133). Unique mutations are also found in the N-terminal region of N which translates into a more significant inhibition of RNA-induced IFN expression (134). Overall, while the functional effect of these mutations has not fully been studied, it is believed that they can modulate host-virus interactions and thus, increase SARS-CoV-2 Omicron replication, pathogenicity, and fitness (135).

Mutations in non-structural proteins for Omicron may also have a crucial effect on immune regulation, transcriptional regulation and viral pathogenesis. For instance, some mutations take place in NSP1, that binds to ribosomal subunits to stop host protein translation (136), or in NSP3, NSP4, and NSP6 that are responsible for viral budding by modifying the endoplasmic reticulum (ER) into double-membrane vesicles (137, 138). In addition, mutations in NSP14 can cause post-transcriptional modifications (139). Mutations have also been observed in NSP5, the main viral protease which also harbors the binding site for this enzyme, and that might which affect viral enzymatic processing activity. Additionaly, mutations detected in NSP6 could help virus survival through the avoidance of autophagosome fusion with lysosomes (140).

Accessory proteins normally act as virulence factors mainly through immune evasion mechanisms that increase viral survival in the host. In SARS-CoV-2, there are eleven accessory proteins of which some are known to be potent interferon antagonists. For instance, in the Omicron variant some mutations have appeared in ORF3a and ORF7b that inhibit STAT signaling phosphorylation and ISGs expression (141, 142). Other examples include ORF3b, ORF6, ORF7a, ORF8 and ORF9b, that are also known to have IFN-antagonistic activity (143–145). ORF9b and ORF9c are also known to interact with cellular organelles, reducing antiviral responses (129, 144–146).

Altogether, it has been shown that Omicron can evade the host immune response more efficiently than previous VOCs. This is credited to decreased recognition by neutralizing antibodies but also to new acquired mutations that lead to increased viral fitness, higher transmission rates and better host immune evasion amongst others.

### 3.2 Nipah virus

Nipah virus (NiV) is an enveloped virus with an 18 kb negative-sense single-stranded RNA genome that belongs to the *Paramyxoviridae* family (147). There are two different strains: NiV Malaysia (NiV-M) and NiV Bangladesh (NiV-B) (148).

NiV outbreaks are reported almost yearly, the most recent occurring in India in 2021, notably with one of the highest fatality rates (92%) observed in the last few years (149). NiV infection in humans is generally associated with an acute respiratory and neurological syndrome resulting in a high fatality rate of between 40% and 92%, depending on the local capacity for epidemiological surveillance and clinical management (150–152).

The NiV reservoir has been identified as fruit bats of the *Pteropus* genus (153). It is known that NiV can also cause severe disease in domestic animals such as pigs, resulting in significant economic losses for farmers (154). Currently, there are no approved treatments or vaccines available for either humans or swine infected with NiV.

Here we aim to summarize the main features of the innate and the adaptive immune response to NiV and discuss the identification of potential immune CoP.

#### 3.2.1 The role of cytokines in NiV infection

NiV infection triggers a robust inflammatory and IFN-I response involving the expression of various IFN-induced antiviral genes. However, to counteract this, NiV expresses several structural and non-structural proteins that can efficiently antagonize a host immune response (155). For instance, the structural matrix M protein and non-structural accessory proteins C, V and W play important roles in preventing IFN-I activation and production at many stages of the signaling pathways involved [summarized in (156)]. In addition, the disproportionate production of pro-inflammatory cytokines at the very early steps of NiV infection in humans considerably contributes to its pathogenicity by causing vasculitis and encephalitis characterized by inflammatory cell infiltration (150).

In this regard, neutrophils are very important during the early steps of the innate immune response since they are involved in several defense mechanisms, including the production of antimicrobial peptides or ROS-induced neutrophil extracellular traps (NETs). However, while in general, NETs can trap and act upon viral particles, in some respiratory virus infections, such as with respiratory syncytial virus (RSV) and influenza A virus (IAV), an exacerbated release of cytokines can lead to high levels of neutrophil activation and excessive NET formation leading to airway occlusion and increased lung inflammation that is detrimental for the host (157). While this has not yet been specifically shown for NiV infection, it is very likely that, similar to what occurs in IAV and RSV infection, a strong release of proinflammatory cytokines leads to hyper-activation of neutrophils which can result in tissue damage. Moreover, it has previously been shown that during NiV infection, while neutrophils do not seem to be infected, they play a prominent role in disseminating NiV (158).

Some of the pro-inflammatory mediators released during NiV infection in humans include TNF-α, CXCL10 and interleukin-1β (IL-1β). The three have been shown to have an important role in disrupting the blood-brain barrier (BBB) and contribute to the neurological symptoms observed in severe cases of NiV disease (NiVD) (150, 157, 159). In addition, other pro-inflammatory mediators such as IL-6, IL-8, MCP-1, GM-CSF and G-CSF have been shown to be released at high levels in severe cases of NiV infection, particularly in the lungs. It has also been shown that this increase in inflammatory chemokines correlates with increased monocyte and T lymphocyte chemotaxis (155).

As a result, it is believed that IFN-I impairment and the release of high levels of pro-inflammatory mediators can contribute to the worsening of clinical symptoms (157). Altogether, data indicates that NiV employs many strategies to counteract the innate immune response and that specific levels of IFN-I and pro-inflammatory cytokines could be used to determine the outcome of the infection. Further study is needed in order to establish these correlations more specifically.

#### 3.2.2 The role of T cell immunity in NiV infection

To date, very little information is available on human cellular immune responses to NiV infection. During the 2018 NiV outbreak in Kerala, India, 18 patients were confirmed to be infected with NiV, of which 2 survived the disease. Cell mediated and humoral immune responses were studied during the acute and convalescent phases of the disease (160). Throughout these periods, surviving patients presented stable T lymphocyte absolute numbers and CD4+ T cells were not more activated than in healthy individuals. However, this was not the case for CD8+ T cells that were more activated and indicated active proliferation and effector functions during the acute phase of the illness and returned to basal levels during the convalescence phase.

Interestingly, the clearance of NiV from the blood seemed to happen before the humoral response (NiV-specific IgG antibodies) took place and rather coincided with the aforementioned activation of CD8+ T cells. In a study where African green monkeys (AGM) were infected with NiV, analysis of the peripheral immune response also showed high levels and activation of T CD8 effector memory cells in surviving AGMs, correlating with an increased release of cytokines and associated cell-mediated immunity (161). This is interesting since CMI was not shown to be relevant in animals that succumbed to NiV infection, thus suggesting that effector memory cells were only relevant in survivors. Interestingly, the activation and proliferation of CD8+ T cells was also observed in the only two survivors of the NiV outbreak in Kerala (160). In contrast, in another study performed in a porcine model, a reduction of CD4+ T cell populations was shown in individuals with a poor clinical outcome (162).

There are several limitations that make it difficult to draw conclusions about cellular immune responses during NiV infection. In human studies, small sample sizes and the lack of samples from disease victims to compare with survivor samples often limit the robustness of the conclusions drawn. Moreover, a complete overview of the relevance of cellular immune responses during NiV infection is lacking, in part due to an absence of CMI response studies in NiV animal models.

During infection, robust T cell responses would enable the development of a faster transition between innate and adaptative immune responses and thus accelerate the production of antibodies and protective immunity. While more data is required, preliminary studies in humans and non-human primates indicate that cellular immune responses, specifically CD8+ T cell activation, seem to be important for protection and therefore CoP for NiVD can be derived from CD8+ T cell measurements.

#### 3.2.3 The role of B cells in NiV infection

Similarly, humoral immune response studies of NiV infection in humans are very limited. However, in the previously mentioned study on the two Kerala NiV survivors in 2018, both patients showed an increased number of B lymphocytes that correlated with the presence of NiV-specific IgG and IgM antibodies within a week after exposure. Moreover, an increased level of activated B cells and plasmablasts was present in both survivor patients (160). However, the specific NiV antigens targeted by the NiV-specific humoral response are yet to be identified.

The correlation between protection against NiV and the presence of antibodies has also been demonstrated in several animal models of infection. For instance, in NiV-infected swine, neutralizing antibodies were detected a week post-infection, with considerably increased titers observed two weeks post-infection. However, NiV RNA could still be detected several months after the initial infection (163). In an AGM model, B cell numbers decreased at twelve days post-infection, a fact that correlated with disease progression and a detrimental outcome (161). In contrast, the only surviving animal in the study showed robust IgM and IgG responses which correlated with an increase in B cell lymphocytes, suggesting that humoral immune responses are relevant during NiV infection and may afford protection against the virus. Moreover, humoral immunity relevance during NiV infection has also been shown in several models including ferrets, hamsters and again in AGM. In these models, the administration of sera or NiV-specific monoclonal antibodies was shown to protect from NiV challenge (164–168).

With regards to fatal cases, there is almost no data for the acute phases of NiVD in humans, and the existing data derives mostly from histopathological analysis of post-mortem samples. However, it has been shown in AGM that lymphopenia takes place in fatal cases of infection with both NiV-M and NiV-B strains (169).

Despite the limitations in human and animal model studies for NiV infection, humoral immune responses seem to play an active role in protection and it is likely that CoP could be derived from humoral immunity parameters such as numbers of plasmablasts and activated B-cells and specific titers of IgM and IgG antibodies.

The main NiVD immune CoP candidates are summarized in **Supplementary Table 1**.

#### 3.2.4 NiV interplay with immune responses

NiV has several proteins that modulate the host immune response. For instance, NiV viral proteins P, C, V and W can antagonize the IFN signaling response (170). While NiV-W protein sequesters STAT1 in the nucleus to inhibit subsequent ISG activation, NiV-V protein antagonizes IFN by binding STAT1 and STAT2 thus preventing their dimerization and transport to the nucleus for transcriptional activation of ISG genes. NiV-P protein is also able to bind and sequester STAT-1 in the nucleus (171, 172). NiV-C protein prevents IFN production in the cytoplasm, but the details of this process are still not well known (156, 173). Further IFN antagonistic mechanisms of *P* gene products are produced through interactions with TANK-binding kinase 1 (TBK1), Inhibitor of κB kinase ϵ (IKKϵ) and IRF-3 by the NiV-W protein (174, 175) or through inhibition of STAT2 (176), LGP2, RIG-I (177), and MDA5 (178) by NiV-V, thus preventing downstream signaling.

Besides *P* gene products, NiV matrix protein (NiV-M), can also inhibit IFN-I. When NiV-M interacts with TRIM6, it promotes its degradation and reduces IKKϵ polyubiquitination thus reducing IFN-mediated responses (179). Moreover, NiV nucleoprotein N can either directly prevent STAT nuclear import or hamper STAT-complex formation, thus also reducing STAT nuclear accumulation and inhibiting type I and II IFN responses (180).

### 3.3 EBOV virus

The genus Ebolavirus contains six virus species, namely *Zaire ebolavirus* (EBOV), *Sudan ebolavirus* (SUDV), *Taï Forest ebolavirus* (TAFV), *Bundibugyo ebolavirus* (BDBV)*, Reston ebolavirus* (RESTV) and *Bombali virus* (BOMV). Out of the six, EBOV is the most prominent member having caused many highly lethal outbreaks in the past. EBOV is a single-negative stranded RNA virus from the *Filoviridae* family (181) and is highly pathogenic for humans and non-human primates. There have been many EBOV outbreaks with high morbidity and mortality since 1976, the 2014 outbreak in West Africa being the deadliest, with more than 28000 recorded cases and 13000 fatalities (182). Due to the multiple transmission mechanisms of EBOV, the broad cellular tropism of the virus and the multiple mechanisms used by EBOV to evade human immune responses, EBOV is considered a highly infectious, category A pathogen. EBOV can cause a highly pathogenic disease, known as Ebola Virus Disease (EVD), with a fatality rate of up to 90% in humans. Cases of EVD are often associated with a septic-shock-like syndrome characterized by an exacerbated inflammatory immune response and coagulopathy, that when combined, lead in many cases to multiple organ failure and death (183).

Whilst many treatments that have been tested in animal models and several vaccines have been shown to induce a very promising immune response against EBOV infection in animal models and humans (some of which are licensed for human use), it is not yet completely clear what are the main protective mechanisms of a successful immune response against EBOV. Although virus neutralizing antibodies and enumeration of polyfunctional T cells (IFN-γ, TNF-α, IL-2) have been associated with protection against EBOV, more data is required to validate these as reliable CoP (184–187). Determining more accurate CoP could facilitate the development of novel, better targeted treatments and vaccines.

#### 3.3.1 The role of cytokines in EBOV infection

EBOV has a broad cellular tropism, with monocytes, dendritic cells and macrophages all being primary cellular targets of the virus. After becoming infected by EBOV, these cells have a pivotal role in the systemic dissemination of the virus through the blood and the lymphatic system. Moreover, their infection also triggers the release of inflammatory mediators such as IL-16, TNF-α, MIP-1α, IL-1β, IL-6, IL-10, amongst others (188, 189). Specifically, high levels of IL-10 and TNF-α are believed to correlate with fatal outcomes from EVD (190, 191).

The virus glycoprotein (GP) and soluble viral proteins such as shed GP are released from infected cells into the extracellular medium, where they have been shown to contribute to the release of proinflammatory cytokines, however, the exact mechanisms responsible for the early cytokine storm are yet to be determined (192, 193).

While monocytes, macrophages and dendritic cells are the main producers of proinflammatory products, other cells such as T cells and endothelial cells are also involved in the release of multiple inflammatory mediators. This results in an immunological disbalance that is believed to, in part, contribute to the severity of EVD.

Overall, the immune disbalance observed during EVD has been shown to be a crucial factor in determining disease severity, since fatal cases often present an exacerbated immune response while survivors, in contrast, mostly display a well-regulated inflammatory response (194).

Other soluble mediators that appear to be extremely relevant during EVD include ROS and IFN I. In the case of IFN I, EBOV VP35 and VP24 proteins act as IFN I transcription and signaling antagonists, respectively (195, 196). Thus, while an early and short-lived production of Type I IFN has been associated with a survival outcome, the absence of IFN is believed to contribute to EBOV dissemination (197). In contrast, an early IFN-γ response followed by lymphopenia is believed to correlate with fatal cases of EVD (188, 190).

ROS has also been shown to have a relevant role in EBOV pathogenesis. For instance, high levels of nitric oxide (NO) are associated with mortality in infected patients (198). Abnormal NO levels are believed to contribute to several pathological disorders such as tissue damage, lymphocyte apoptosis and the disruption of vascular integrity.

There are several coagulopathies associated with EVD such as thrombocytopenia or the presence of high levels of fibrin degradation products. In some cases, this leads to DIC, which frequently contributes to multiorgan failure (199). While not all of the mechanisms responsible for triggering EBOV-related coagulopathy are fully understood, the results of several studies strongly suggest that the exacerbated release of proinflammatory mediators considerably contributes to these characteristic EVD coagulopathies. For instance, the hyperproduction of proinflammatory cytokines activates coagulation factors such as procoagulant protein tissue factor (TF), fibrin fragment E and thrombin, which in turn, upregulate the production of proinflammatory cytokines (190, 200).

It has also been observed that endothelial cells are severely affected in late stages of EVD. Due to exceedingly high levels of proinflammatory cytokines (ROS and TF amongst other soluble mediators) endothelial cells are activated and endothelial leakage occurs (201).

Therefore, upon EBOV infection, a chain reaction initiated by an exacerbated inflammation response leads to a disbalanced immune response, systemic virus spread, vascular damage and coagulopathies that altogether will lead to a septic-shock like syndrome and multiorgan failure.

#### 3.3.2 The role of T cell immunity in EBOV infection

Although EBOV does not infect lymphocytes, it can interact with T cells, affecting the development of immune responses. T-cell mediated immune responses during EVD involve a robust activation of T cells followed by their proliferation in both fatal cases and survivor patients (202). The magnitude and diversity of T-cell mediated immune responses in survivors during EVD are more robust when compared to fatal cases. In fatal cases there is an early T cell activation followed by a T cell population collapse, probably due to T-cell exhaustion (203, 204). Moreover, oligoclonal T-cell responses and higher expression of T cell inhibitory molecules CTLA-4 and PD-1 in CD8+ and CD4+ T cells are believed to contribute to an inefficient T cell response in fatal cases that is associated with higher viral loads when compared to survivors (205, 206). In this regard, the early cytokine storm observed in fatal cases correlates with high later expression levels of CTLA-4 and PD-1 in T cells (206). In contrast, survivors would appear to develop a very diverse T cell response with low levels of CTLA-4 and PD-1 T cell inhibitors, thus contributing to viral clearance. However, a more recent study has shown that West African EVD survivors from 2013-2016, presented an increase in activation and proliferation markers in CD4+ and CD8+ T cell populations by 30% and 50% respectively, when compared with healthy individuals (202). This increased activation and proliferation suggests that survivor patients can develop a robust immune response. This activation was shown to last more than one month after recovery.

Interestingly, during the EVD convalescent phase, CD4+ and CD8+ T lymphocytes from survivor patients were able to respond to EBOV nucleoprotein (NP) thus indicating that EBOV NP can stimulate virus-specific-T cell responses in humans after resolution of the disease. Similarly, in another study in EVD patients from the 2013-2016 outbreak, it was shown that survivor memory CD8+ T cells can secrete IFN-γ and TNF-α and mainly responded to viral NP and to a lesser degree to VP24, VP40, VP35 and GP. This data would appear to corroborate the immunodominance of the EBOV NP-specific T cell responses described in previous studies (205). Studies in mice, guinea pigs and NHP models have also highlighted the importance of T cell responses during EVD and the involvement of the viral NP in generating T-cell immunity (207–209).

In cases of EVD, lymphocytes are severely affected and undergo apoptosis thus making lymphoid depletion a prominent feature of the disease (208, 210). In fatal cases, there is approximatively one fourth less lymphocytes when compared with levels found in survivors (190). This loss of lymphocytes is believed to be due to several factors, including the combined impairment of DC associated with the previously mentioned abnormal release of inflammatory cytokines, including TNF-related apoptosis-inducing ligand (TRAIL) and Fas death receptor, upon EBOV infection (194, 211). Abnormal levels of NO and direct interactions between EBOV and lymphocytes are also believed to contribute to the loss of bystander lymphocytes during infection (183, 212). In addition, it has also been shown that phosphatidylserine associated with EBOV GP can bind and stimulate CD4+ T cells through T-cell immunoglobulin mucin receptor 1 (TIM-1). These cells then release proinflammatory mediators believed to contribute to the cytokine storm and the lymphopenia observed during EVD (213). Other studies have determined that abortive infection of T lymphocytes causes ER-stress in these cells thus contributing to their own apoptosis (214). Importantly, lymphopenia was shown to correlate with fatal cases during the 2000 Ebola Sudan outbreak in Uganda (198).

To summarize, the proliferation of lymphocytes is observed in both survivors and fatal human cases, however in the latter, T lymphocytes display less immune response diversity and frequently show lymphopenia in the later stages of EVD. While it has been determined that robust T cell mediated immune responses can be a CoP during EVD, it is clear that immune responses are not independent compartments and the appreciating the interplay between innate and adaptive immunity may be crucial in understanding the complexity required to produce an efficient protective immune response during EVD.

#### 3.3.3 The role of B cell immunity in EBOV infection

Even though T cell mediated immune responses are crucial during EVD, humoral immune responses also play a very relevant role in EBOV clearance. It has previously been shown that survivors tend to produce early and sustained levels of IgG, while fatalities have rather an impaired humoral response characterized by the absence of EBOV-specific IgG, low levels of IgM and lymphopenia (215, 216). Upon EBOV infection, IgM can be detected as early as day 2 after symptoms appear and in the case of IgG, antibodies are normally present between days 5 to 18 days after symptom onset (202, 217, 218). After one year of symptom onset, an IgM repertoire against VP40 and GP was observed in survivors despite undetectable virus levels. This however could also be an indicator of hidden viral persistence (194). Interestingly, serological surveys of IgG levels in rural villages in Gabon showed EBOV antibody seroprevalence, suggesting either prior exposure to EBOV or the presence of cross-reactive antibodies (219).

It is however unclear how long the immunity in EBOV survivors lasts; in some cases, it has been shown that EBOV-specific antibodies are present for forty years after symptomatic infection (220). These antibodies have been shown to have pan-neutralizing capacity against EBOV *in vitro* and were associated with protective roles in several animal models such as mice, guinea pigs and ferrets (221–225). However, whether these antibodies have a protective potential against EBOV reinfection in survivors remains undetermined. It should be considered that EBOV neutralization may not always translate into protection in humans and frequently, other antibody functions (complement, opsonization…) have been shown to be important in surviving EVD (226, 227).

In order to assess serological immune profiles of EVD survivors, antibody isotypes were analyzed and showed changes in the antibody repertoire over time. While neutralizing EBOV-specific IgG1 persisted over time, IgG3 decreased in early phases and IgG4 appeared later on. Moreover, IgA with innate immune effector functions and long-lasting IgG/IgM/IgA epitope diversity were described in EVD survivors (228, 229).

Not all of the antibodies detected can recognize EBOV GP. Survivors from the 1976 Yambuku outbreak for example have been shown to harbor antibodies with reactivity to GP, NP and to a lesser extent VP40. However, all identified antibodies with neutralizing capacity were GP-specific in humans and animal models (220, 230, 231). It is for this reason that most vaccines are based on EBOV GP.

Antibodies can target nearly any region on the surface of EBOV GP. Conserved GP regions include the receptor binding site (RBS), the base, the internal fusion loop (IFL) and the heptad repeat 2 (HR2). Other regions such as the glycan cap region and mucin-like domain (MLD) are less conserved (232). While conserved regions are normally targeted by cross-reactive antibodies, most of the antibody responses found in survivors target less conserved regions since they are structurally more exposed. However, these antibodies are frequently non-neutralizing, show weak affinity and are non-cross-reactive (233). There are nevertheless some antibodies that target the glycan cap and are pan-protective, neutralizing several EBOV species (234) Recently, a conserved site named the MLD cradle that connects the MLD to the glycan cap has been identified as an antibody target region that destabilizes the GP quaternary structure, blocking the receptor binding required for effective EBOV infection (235). This is an important step forward in determining the molecular basis of EBOV neutralization by targeting conserved exposed epitopes and could be used to design universal antibody therapeutics.

There are currently two approved vaccines against EBOV, namely rVSV-ZEBOV (Ervebo) and ChAd3-MVA (Ad26) (236, 237). They have both been shown to induce EBOV-specific humoral and cellular responses, however these immune responses are not identical to the ones observed in survivors. In order to generate efficient vaccines, it is thus important to compare immunogenicity and protection between vaccinees and survivors. For instance, there are serological studies of immune memory responses showing that EVD survivors (2-6 months after infection) from the 2013-2016 EBOV outbreak have higher antibody levels and stronger antibody affinity when compared to ChAd3-MVA vaccinees at 2-12 months. Moreover, while this cohort of vaccinees had a predominant IgM response, survivors displayed a higher level of IgG with a more diverse antibody repertoire than the vaccinees (230, 238). Interestingly, survivor antibodies were shown to preferentially target the fusion peptide and HR2 domains of the viral GP2 protein and provide neutralization (238).

In the case of rVSV-ZEBOV, a serological study comparing survivors versus rVSV-ZEBOV vaccinees showed that survivor IgM and IgG do not bind the same EBOV GP epitopes when compared to rVSV-ZEBOV vaccinees (239). Additionally, another study in a similar cohort showed no significant differences in circulating antibody subclass levels. However, survivor antibodies had a higher neutralization capacity and a higher capacity to induce cellular responses than those from vaccinee samples. Importantly, IgG1 levels in survivors correlated with EBOV neutralization capacity, which was not the case in vaccinees (240).

These studies provide a good overview of the potential differences between survivor and vaccinee immune responses and will surely contribute to the development of more efficient next-generation vaccines. The main EVD immune CoP candidates are shown in **Supplementary Table 1**.

#### 3.3.4 EBOV interplay with immune responses

EBOV has two main strategies to interfere with the host immune response. First, EBOV blocks IFN signaling and production through VP24 and VP35, respectively. This way, both proteins together ensure that IFN production is hampered and in the case that IFN is produced, the infected cell is unable to respond (241). In the case of VP24, this protein can either directly bind to STAT-1 thus blocking its transport to the nucleus or it can bind to karyopherin α1 and consequently block the IFN antiviral response (242, 243). EBOV VP35 on the other hand, antagonizes IFN mainly through blocking Interferon regulatory factor 3 (IRF-3) phosphorylation and protein kinase R (196, 244).

The second mechanism used by EBOV for immune diversion involves several glycoproteins. The EBOV fourth gene encodes for three different glycoproteins depending on the number of uracyls (Us) added at a so-called editing site. The viral structural surface glycoprotein (EBOV GP) is transcribed when 8 Us are found the editing site (245, 246). When 7Us and 6U/9Us are present, different soluble glycoproteins are produced, namely secreted glycoprotein (sGP) and small soluble GP (ssGP) respectively. Moreover, an additional soluble glycoprotein is generated when a percentage of the EBOV GP expressed on the surface of infected cells is cleaved by proteases releasing it in a soluble form with no transmembrane domain, known as shed GP. Due to its structural similarity to EBOV GP, it has been suggested that shed GP has a role in recruiting new primary targets and also binds antibodies directed to the virus (192). While not all functions have clearly been elucidated, it is suggested that soluble glycoprotein sGP may also play a role in immune evasion by binding antibodies initially directed against the viral surface glycoprotein EBOV GP. However, since not all amino acids are identical to the surface glycoprotein, it is believed that sGP also acts as decoy antigen and reduces specific antibody production against surface GP, possibly resulting in antigenic subversion (247). Importantly, sGP also exhibits anti-inflammatory activities in the endothelium and by reducing the amount of CD16b receptor on human neutrophils thus preventing their activation and consequently stunting an innate immune response (248). Regarding ssGP, while it is believed that it could share some of the functions described for sGP (249), its specific role during EBOV pathogenesis has not yet been clearly elucidated.

## 4 Comparison of immune profiling in SARS-CoV-2, NiV and EBOV infection

To effectively tackle emerging viruses, it is essential to understand the potential similarities and differences between virus families, the viruses themselves and the immune responses that they elicit upon infection in order to establish realiable CoP. SARS-CoV-2, NiV and EBOV share some immune signatures, yet currently not all of the molecular mechanisms involved in fighting infection with these viruses have been elucidated. These emerging viruses all have in common that they dysregulate host immune responses, including both early and late events, and this dysregulation is associated with viral progression during COVID-19, NiVD and EVD. Below we provide a comparison of the key features of immune responses to SARS-CoV-2, NiV and EBOV infection in humans (**Table 1**), to better understand differences and the potential pathways to derive CoP (or pathology) for the three diseases.

**Table 1:** Key features of immune responses to SARS-CoV-2, NiV and EBOV infecCon in humans.

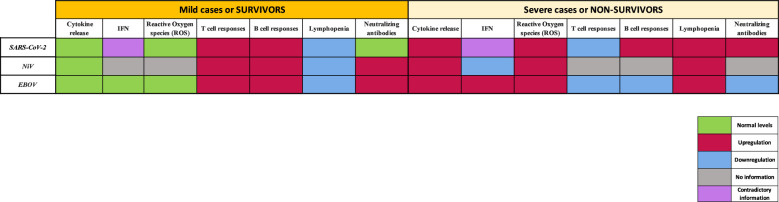

The exacerbated release of cytokines and chemokines plays a major role in SARS-CoV-2, NiV and EBOV immunopathology, as this can eventually lead to severe complications and in some cases death during all three diseases. Moreover, abnormal inflammation levels have a big influence in late-stage cellular and humoral immune responses. In this regard, the level of activation, proliferation, phenotype and kinetics of T lymphocyte populations can direct specific T cell mediated and humoral responses and influence the severity of the aforementioned emerging diseases.

The presence of neutralizing IgG in serum is in general used as a CoP, however the titer and the type of antibodies that are needed to reach protection for each disease (or disease outcome) are not clear and need to be defined more precisely and more specifically for each manifestation of infection (protection against infection, protection against death or severe disease, chronic infection, etc…). The kinetics of humoral responses and additional antibody functions such as ADCC may also play a crucial role in protection. Moreover, currently, most vaccines are focused on the glycoproteins of the virus envelope as the immunogen, however, as described above, the viral nucleoproteins and other non-envelope proteins should also be considered in future vaccine designs, and consequently the definition of CoP be updated.

Taken in combination, all of these factors highlight the importance of the different immune compartments and their interactions in achieving viral clearance and highlight the need to further understand innate, cellular and humoral responses and their interplay in order to identify more specific CoP. It is also important to consider the relevance of the potential differences amongst host immune responses during disease progression and to appreciate the role of host diversity in determining the ability to survive infection. The evaluation of common immune signatures that lead to the transition from a mild disease state to a severe one will help in finding novel preventive measures and treatments that could reduce mortality rates.

## 5 Conclusions

In summary, defining which immune effector mechanisms play a role in protective immunity is crucial for the rational design of vaccines and therapeutics and also for deriving CoP. The latter could be used as surrogates of protection so that evaluation of the protective efficacy of new medical countermeasures can be facilitated. This will in turn guide the pathway for the acceleration of the licensing of these products by regulatory agencies.

For certain pathogens, when survival rates are low, it is not easy to define the underlying immune mechanisms that correlate with protection. The lack of a full understanding of natural immune responses and the potential of certain pathogens to evade them complicates the derivation of CoP. The immune response components described above are an example of the variety of the different and non-exclusive mechanisms that the human immune system uses to evoke the desired protective immunity. Moreover, these mechanisms are not always systemic and for instance, there are often organ-specific mechanisms of immunity (mucosal immunity). More research towards the definition of organ-specific protective mechanisms would help in determining more reliable CoP for certain diseases and vaccines.

The nature and complexity of the interactions of the different cells and soluble effectors of the immune system that are involved in protection is remarkable. Indeed, humoral and cell-mediated immune responses do not act in isolation and the innate immune response strongly influences both T-cell mediated and humoral responses. The best protection against most pathogens is achieved when both arms of the immune system act cooperatively in synergy. While pre-existing antibodies and natural immunity mechanisms may provide the first line of adaptive immune defense, when it is breached, memory T and B cell responses come into play. Thus, an ideal vaccine should offer an integrative approach that triggers both protective antibody levels with robust immunological memory and rapid and efficient effector functions. The high degree of variability of surface antigens in certain pathogens and the complex and dynamic nature of host-pathogen interactions, render the development of vaccines against intracellular infections a challenging process. Notably, such infections often also require cell‐mediated immunity.

A limitation of current vaccine development strategies however, is the reductionist approach of measuring vaccine efficacy as a function of measurable antibody responses, which are often used as CoP, mainly for reasons related to ease of detection, quantification and ease of standardization. Whilst this strategy has proven useful for certain vaccines, it has also shown limitations. This is exemplified by the case of using a serum antibody titer of 1/40 in hemagglutination inhibition tests as a CoP for Influenza vaccines. This way of assessing protective immunity is becoming obsolete as further studies have revealed that different population sub-groups (i.e. the elderly and children) require different titers for predicting protection, particularly as new vaccine strategies for flu based on viral vectors (including other antigens in addition to HA) and mucosal delivery routes (which induce different type of immune effector mechanisms) become available. In the case of EBOV, NiV and SARS-CoV-2, virus neutralizing antibodies have been used as CoP, but again as explained above, this parameter has its own limitations as it is clear that other arms of the immune system do play a role in protection that may not necessarily correlate with neutralizing Ab levels. Furthermore, it is important to emphasize that CoP need to be defined for a specific set of conditions that are related to host, population group, specific disease manifestation that the vaccine intends to protect against and dose, amongst other factors. The large variation in immune responses of the host and the heterogeneity in terms of genetics, age, sex, individual variation and environmental factors, including previous infection status, adds to the challenge of obtaining efficient vaccines.

For all the reasons described above, gaining a deeper understanding of the underlaying immune mechanisms and requirements for successful outcomes during infection is essential in order to derive CoP that are accurate and reliable. This would translate to the development of more effective vaccines and provide more confidence in the ways in which these vaccines are assessed. Vaccine efficacy could be dramatically improved by targeting specific immune CoP such as the generation and maintenance of distinct memory T cell subsets, the specific release of cytokines or facilitating the production of neutralizing antibodies.

The increasing focus on characterizing immune responses to viral infections has led to the development of novel approaches to detect common immune features conferring protection. This has resulted in the development of *in silico* prediction targets that ultimately may result in the definition of CoP against prominent current pathogens but also for future emerging ones. From a long-term perspective, understanding immune CoP that are specific for certain pathogens could help to promote long-term immunological health. Hence, at a time when emerging infections seem to be more and more frequent, the speed of the efficient establishment of immune CoP appears to be a critical factor in the fight against present and future health threatening diseases.

## Author contributions

Conceptualization was done by BE-P and JC-O; writing—original draft preparation, BE-P, PL and JC-O; writing—review and editing, BE-P, PL and JC-O. All authors contributed to the article and approved the submitted version.
