# Supplementary material for: Immune correlates of protection for SARS-CoV-2, Ebola and Nipah virus infection
Source: Front Immunol. 2023 Apr 17;14:1156758. doi: 10.3389/fimmu.2023.1156758 (PMC10158532; doi:10.3389/fimmu.2023.1156758)
Supplement: Supplementary file 1 [file Table_1.docx]

**Supplementary Table 1.** **Candidate CoP for COVID-19, NiVD and EVD.**

Candidate CoP for COVID-19, NiVD and EBV are indicated in blue. The immune correlates that are not considered as CoP candidates but are present in severe or lethal cases are indicated in red. In grey, the immune factors that have a non-clear role or that are not yet fully described in the literature.

|  |  | **Biomarker**  **category levels** | **COVID-19** | **NiVD** | **EVD** | **References** |
| --- | --- | --- | --- | --- | --- | --- |
| **Innate Immune responses** | **Humoral** | Proinflammatory cytokines |  |  |  | (1-5)  (6-8)  (9-12) |
|  |  | Complement  (CDC) |  |  | c.i. | (13, 14)  (15, 16)  (17-19) |
|  |  | Natural antibodies | n.i.a. | n.i.a. | n.i.a. | -  -  - |
|  |  | Pentraxins | PTX3 | n.i.a. |  | (20, 21)  -  (22) |
|  | **Cellular** | Phagocytes |  |  |  | (23-26)  (27, 28)  (11, 29, 30) |
|  |  | Mast cells |  | n.i.a. | n.i.a. | (31-33)  -  - |
|  |  | Basophils and eosinophils | basophils |  | n.i.a. | (34-36)  (37-42)  - |
|  |  |  | eosinophils |  |  |  |
|  |  | γδ T cells |  |  |  | (43-45)  (46)  (47) |
|  |  | NK cells | * |  |  | (48-53)  (42, 46)  (54-56) |
| **Adaptive Immune responses** | **Humoral** | Neutralizing Ab ^1^ | ** |  |  | (57-62)  (42, 63-72)  (73-79) |
|  |  | Surface viral protein Ab ^1,2^ | Anti-S | Anti-G  Anti-F | Anti-GP | (80, 81)  (70-72, 82, 83)  (79, 84) |
|  |  | Nucleocapsid Ab | Anti-N ^2,3^ | n.i.a. | Anti-NP ^2^ | (80, 81, 85)  -  (75, 79) |
|  |  | Fc-FcR Functions | ADCC ADCP | ADCC ADCP  (IgG2c) | ADCC ADCP | (13, 86-88)  (89)  (18, 90) |
|  |  | B-cell Memory | IgG1, IgG3 | IgG1 | IgG1, IgA1 | (57-59, 91, 92)  (42, 46, 93)  (79, 94) |
|  |  | Mucosal Ab | IgA | IgA | IgA | (95-99)  (100)  (79, 101) |
|  | **Cellular** | T-cell Phenotype | Th1, Polyfunctional  T cells  (IFNγ, TNFα, IL-2) | Activated CD8+ effector T cells  (Ki67^+^, PD-1 granzyme B) | Dominant CD8+ polyfunctional T cell  (IFNγ, TNFα and IL-2) | (102-104)  (8, 42, 63, 105)  (106, 107) |
|  |  | T-cell memory | CD4+ and CD8+ T cell memory | CD8+ effector memory T cells | Central memory CD4+ T cells | (91, 104, 108)  (42, 63)  (106, 107) |
|  |  | Mucosal T-cell responses | CD4+ and CD8+ T cell | n.i.a. | *** | (109)  -  (110, 111) |

Ab: Antibody; ADCC: Antibody-Dependent Cellular Cytotoxicity; ADCP: Antibody-Dependent Cellular Phagocytosis; CDC: Complement-dependent cytotoxicity; c.i.: contradictory information; n.i.a.: no information available; PTX3: pentraxin 3

*: protection applies only to certain NK subtypes; long-term COVID-19 patients seem to have high levels of NK cells.

**: protection varies depending on the VOCs and the measurement time after infection/immunization.

***: only fatal cases present T cell clustering in the gut and the respiratory mucosa.

^1^: the main techniques used to determine the presence of neutralizing Ab include: pseudotype neutralization assay (pMN) and virus neutralization (VN) test.

^2^: the main techniques used to determine the presence of antibodies include Enzyme-linked Immunosorbent Assay (ELISA) and Luminex.

| **CoP** |  |
| --- | --- |
| **Non-CoP** |  |
| **Contradictory information or not yet described** |  |

^3^: neutralizing capacity takes place through Trim-21 mediated Intracellular Neutralizing antibodies (T21VNAb) and was quantified by electroporated antibody-dependent neutralization assay (EDNA).

**References from Supplementary Table 1**

1. Yang Y, Shen C, Li J, Yuan J, Wei J, Huang F, Wang F, Li G, Li Y, Xing L, Peng L, Yang M, Cao M, Zheng H, Wu W, Zou R, Li D, Xu Z, Wang H, Zhang M, Zhang Z, Gao GF, Jiang C, Liu L, Liu Y. Plasma IP-10 and MCP-3 levels are highly associated with disease severity and predict the progression of COVID-19. J Allergy Clin Immunol. 2020;146(1):119-27 e4.

2. Qin C, Zhou L, Hu Z, Zhang S, Yang S, Tao Y, Xie C, Ma K, Shang K, Wang W, Tian DS. Dysregulation of Immune Response in Patients With Coronavirus 2019 (COVID-19) in Wuhan, China. Clin Infect Dis. 2020;71(15):762-8.

3. Arunachalam PS, Wimmers F, Mok CKP, Perera R, Scott M, Hagan T, Sigal N, Feng Y, Bristow L, Tak-Yin Tsang O, Wagh D, Coller J, Pellegrini KL, Kazmin D, Alaaeddine G, Leung WS, Chan JMC, Chik TSH, Choi CYC, Huerta C, Paine McCullough M, Lv H, Anderson E, Edupuganti S, Upadhyay AA, Bosinger SE, Maecker HT, Khatri P, Rouphael N, Peiris M, Pulendran B. Systems biological assessment of immunity to mild versus severe COVID-19 infection in humans. Science. 2020;369(6508):1210-20.

4. Sarzi-Puttini P, Giorgi V, Sirotti S, Marotto D, Ardizzone S, Rizzardini G, Antinori S, Galli M. COVID-19, cytokines and immunosuppression: what can we learn from severe acute respiratory syndrome? Clinical and experimental rheumatology. 2020;38(2):337-42.

5. Fu Y, Cheng Y, Wu Y. Understanding SARS-CoV-2-Mediated Inflammatory Responses: From Mechanisms to Potential Therapeutic Tools. Virol Sin. 2020;35(3):266-71.

6. Ang BSP, Lim TCC, Wang L. Nipah Virus Infection. J Clin Microbiol. 2018;56(6).

7. Mathieu C, Guillaume V, Sabine A, Ong KC, Wong KT, Legras-Lachuer C, Horvat B. Lethal Nipah virus infection induces rapid overexpression of CXCL10. PLoS One. 2012;7(2):e32157.

8. Liew YJM, Ibrahim PAS, Ong HM, Chong CN, Tan CT, Schee JP, Gomez Roman R, Cherian NG, Wong WF, Chang LY. The Immunobiology of Nipah Virus. Microorganisms. 2022;10(6).

9. Wauquier N, Becquart P, Padilla C, Baize S, Leroy EM. Human fatal zaire ebola virus infection is associated with an aberrant innate immunity and with massive lymphocyte apoptosis. PLoS Negl Trop Dis. 2010;4(10).

10. McElroy AK, Harmon JR, Flietstra TD, Campbell S, Mehta AK, Kraft CS, Lyon MG, Varkey JB, Ribner BS, Kratochvil CJ, Iwen PC, Smith PW, Ahmed R, Nichol ST, Spiropoulou CF. Kinetic Analysis of Biomarkers in a Cohort of US Patients With Ebola Virus Disease. Clin Infect Dis. 2016;63(4):460-7.

11. Falasca L, Agrati C, Petrosillo N, Di Caro A, Capobianchi MR, Ippolito G, Piacentini M. Molecular mechanisms of Ebola virus pathogenesis: focus on cell death. Cell Death Differ. 2015;22(8):1250-9.

12. Villinger F, Rollin PE, Brar SS, Chikkala NF, Winter J, Sundstrom JB, Zaki SR, Swanepoel R, Ansari AA, Peters CJ. Markedly elevated levels of interferon (IFN)-gamma, IFN-alpha, interleukin (IL)-2, IL-10, and tumor necrosis factor-alpha associated with fatal Ebola virus infection. J Infect Dis. 1999;179 Suppl 1:S188-91.

13. Adeniji OS, Giron LB, Purwar M, Zilberstein NF, Kulkarni AJ, Shaikh MW, Balk RA, Moy JN, Forsyth CB, Liu Q, Dweep H, Kossenkov A, Weiner DB, Keshavarzian A, Landay A, Abdel-Mohsen M. COVID-19 Severity Is Associated with Differential Antibody Fc-Mediated Innate Immune Functions. mBio. 2021;12(2).

14. Afzali B, Noris M, Lambrecht BN, Kemper C. The state of complement in COVID-19. Nat Rev Immunol. 2022;22(2):77-84.

15. Johnson JB, Borisevich V, Rockx B, Parks GD. A novel factor I activity in Nipah virus inhibits human complement pathways through cleavage of C3b. J Virol. 2015;89(2):989-98.

16. Johnson JB, Aguilar HC, Lee B, Parks GD. Interactions of human complement with virus particles containing the Nipah virus glycoproteins. J Virol. 2011;85(12):5940-8.

17. Mellors J, Tipton T, Fehling SK, Akoi Bore J, Koundouno FR, Hall Y, Hudson J, Alexander F, Longet S, Taylor S, Gorringe A, Magassouba N, Konde MK, Hiscox J, Strecker T, Carroll M. Complement-Mediated Neutralisation Identified in Ebola Virus Disease Survivor Plasma: Implications for Protection and Pathogenesis. Front Immunol. 2022;13:857481.

18. Takada A, Feldmann H, Ksiazek TG, Kawaoka Y. Antibody-dependent enhancement of Ebola virus infection. J Virol. 2003;77(13):7539-44.

19. Brudner M, Karpel M, Lear C, Chen L, Yantosca LM, Scully C, Sarraju A, Sokolovska A, Zariffard MR, Eisen DP, Mungall BA, Kotton DN, Omari A, Huang IC, Farzan M, Takahashi K, Stuart L, Stahl GL, Ezekowitz AB, Spear GT, Olinger GG, Schmidt EV, Michelow IC. Lectin-dependent enhancement of Ebola virus infection via soluble and transmembrane C-type lectin receptors. PLoS One. 2013;8(4):e60838.

20. Assandri R, Accordino S, Canetta C, Buscarini E, Scartabellati A, Tolassi C, Serana F. Long pentraxin 3 as a marker of COVID-19 severity: evidences and perspectives. Biochem Med (Zagreb). 2022;32(2):020901.

21. Margiana R, Sharma SK, Khan BI, Alameri AA, Opulencia MJC, Hammid AT, Hamza TA, Babakulov SK, Abdelbasset WK, Jawhar ZH. The pathogenicity of COVID-19 and the role of pentraxin-3: An updated review study. Pathol Res Pract. 2022;238:154128.

22. Cilloniz C, Ebihara H, Ni C, Neumann G, Korth MJ, Kelly SM, Kawaoka Y, Feldmann H, Katze MG. Functional genomics reveals the induction of inflammatory response and metalloproteinase gene expression during lethal Ebola virus infection. J Virol. 2011;85(17):9060-8.

23. Abdelmoaty MM, Yeapuri P, Machhi J, Olson KE, Shahjin F, Kumar V, Zhou Y, Liang J, Pandey K, Acharya A, Byrareddy SN, Mosley RL, Gendelman HE. Defining the Innate Immune Responses for SARS-CoV-2-Human Macrophage Interactions. Front Immunol. 2021;12:741502.

24. Matveeva O, Nechipurenko Y, Lagutkin D, Yegorov YE, Kzhyshkowska J. SARS-CoV-2 infection of phagocytic immune cells and COVID-19 pathology: Antibody-dependent as well as independent cell entry. Front Immunol. 2022;13:1050478.

25. Peyneau M, Granger V, Wicky PH, Khelifi-Touhami D, Timsit JF, Lescure FX, Yazdanpanah Y, Tran-Dinh A, Montravers P, Monteiro RC, Chollet-Martin S, Hurtado-Nedelec M, de Chaisemartin L. Innate immune deficiencies are associated with severity and poor prognosis in patients with COVID-19. Sci Rep. 2022;12(1):638.

26. McKenna E, Wubben R, Isaza-Correa JM, Melo AM, Mhaonaigh AU, Conlon N, O'Donnell JS, Ni Cheallaigh C, Hurley T, Stevenson NJ, Little MA, Molloy EJ. Neutrophils in COVID-19: Not Innocent Bystanders. Front Immunol. 2022;13:864387.

27. Tiong V, Shu MH, Wong WF, AbuBakar S, Chang LY. Nipah Virus Infection of Immature Dendritic Cells Increases Its Transendothelial Migration Across Human Brain Microvascular Endothelial Cells. Front Microbiol. 2018;9:2747.

28. Mathieu C, Pohl C, Szecsi J, Trajkovic-Bodennec S, Devergnas S, Raoul H, Cosset FL, Gerlier D, Wild TF, Horvat B. Nipah virus uses leukocytes for efficient dissemination within a host. J Virol. 2011;85(15):7863-71.

29. Rogers KJ, Maury W. The role of mononuclear phagocytes in Ebola virus infection. J Leukoc Biol. 2018;104(4):717-27.

30. Wanninger TG, Millian DE, Saldarriaga OA, Maruyama J, Saito T, Reyna RA, Taniguchi S, Arroyave E, Connolly ME, Stevenson HL, Paessler S. Macrophage infection, activation, and histopathological findings in ebolavirus infection. Front Cell Infect Microbiol. 2022;12:1023557.

31. Krysko O, Bourne JH, Kondakova E, Galova EA, Whitworth K, Newby ML, Bachert C, Hill H, Crispin M, Stamataki Z, Cunningham AF, Pugh M, Khan AO, Rayes J, Vedunova M, Krysko DV, Brill A. Severity of SARS-CoV-2 infection is associated with high numbers of alveolar mast cells and their degranulation. Front Immunol. 2022;13:968981.

32. Budnevsky AV, Avdeev SN, Kosanovic D, Shishkina VV, Filin AA, Esaulenko DI, Ovsyannikov ES, Samoylenko TV, Redkin AN, Suvorova OA, Perveeva IM. Role of mast cells in the pathogenesis of severe lung damage in COVID-19 patients. Respir Res. 2022;23(1):371.

33. Weinstock LB, Brook JB, Walters AS, Goris A, Afrin LB, Molderings GJ. Mast cell activation symptoms are prevalent in Long-COVID. Int J Infect Dis. 2021;112:217-26.

34. Bonam SR, Chauvin C, Levillayer L, Mathew MJ, Sakuntabhai A, Bayry J. SARS-CoV-2 Induces Cytokine Responses in Human Basophils. Front Immunol. 2022;13:838448.

35. Murdaca G, Di Gioacchino M, Greco M, Borro M, Paladin F, Petrarca C, Gangemi S. Basophils and Mast Cells in COVID-19 Pathogenesis. Cells. 2021;10(10).

36. Tabachnikova A, Chen ST. Roles for eosinophils and basophils in COVID-19? Nat Rev Immunol. 2020;20(8):461.

37. Lo MK, Miller D, Aljofan M, Mungall BA, Rollin PE, Bellini WJ, Rota PA. Characterization of the antiviral and inflammatory responses against Nipah virus in endothelial cells and neurons. Virology. 2010;404(1):78-88.

38. Hammoud DA, Lentz MR, Lara A, Bohannon JK, Feuerstein I, Huzella L, Jahrling PB, Lackemeyer M, Laux J, Rojas O, Sayre P, Solomon J, Cong Y, Munster V, Holbrook MR. Aerosol exposure to intermediate size Nipah virus particles induces neurological disease in African green monkeys. PLoS Negl Trop Dis. 2018;12(11):e0006978.

39. Stevens CS, Lowry J, Juelich T, Atkins C, Johnson K, Smith JK, Panis M, Ikegami T, tenOever B, Freiberg AN, Lee B. Nipah virus Bangladesh infection elicits organ-specific innate and inflammatory responses in the marmoset model. J Infect Dis. 2023.

40. Lam SK, Chua KB. Nipah virus encephalitis outbreak in Malaysia. Clin Infect Dis. 2002;34 Suppl 2:S48-51.

41. Cline C, Bell TM, Facemire P, Zeng X, Briese T, Lipkin WI, Shamblin JD, Esham HL, Donnelly GC, Johnson JC, Hensley LE, Honko AN, Johnston SC. Detailed analysis of the pathologic hallmarks of Nipah virus (Malaysia) disease in the African green monkey infected by the intratracheal route. PLoS One. 2022;17(2):e0263834.

42. Lara A, Cong Y, Jahrling PB, Mednikov M, Postnikova E, Yu S, Munster V, Holbrook MR. Peripheral immune response in the African green monkey model following Nipah-Malaysia virus exposure by intermediate-size particle aerosol. PLoS Negl Trop Dis. 2019;13(6):e0007454.

43. Gay L, Rouviere M-S, Mezouar S, Richaud M, Gorvel L, Foucher E, Madakamutil L, La Scola B, Menard A, Allardet-Servent J, Halfon P, Frohna P, Cano C, Mege J-L, Olive D. Vγ9Vδ2 T cells are potent inhibitors of SARS-CoV-2 replication and exert effector phenotypes in COVID-19 patients. bioRxiv. 2022:2022.04.15.487518.

44. Lei L, Qian H, Yang X, Zhang X, Zhang D, Dai T, Guo R, Shi L, Cheng Y, Zhang B, Zhou X, Hu J, Guo Y. The phenotypic changes of gammadelta T cells in COVID-19 patients. J Cell Mol Med. 2020;24(19):11603-6.

45. von Massow G, Oh S, Lam A, Gustafsson K. Gamma Delta T Cells and Their Involvement in COVID-19 Virus Infections. Front Immunol. 2021;12:741218.

46. Foster SL, Woolsey C, Borisevich V, Agans KN, Prasad AN, Deer DJ, Geisbert JB, Dobias NS, Fenton KA, Cross RW, Geisbert TW. A recombinant VSV-vectored vaccine rapidly protects nonhuman primates against lethal Nipah virus disease. Proc Natl Acad Sci U S A. 2022;119(12):e2200065119.

47. Cimini E, Viola D, Cabeza-Cabrerizo M, Romanelli A, Tumino N, Sacchi A, Bordoni V, Casetti R, Turchi F, Martini F, Bore JA, Koundouno FR, Duraffour S, Michel J, Holm T, Zekeng EG, Cowley L, Garcia Dorival I, Doerrbecker J, Hetzelt N, Baum JHJ, Portmann J, Wolfel R, Gabriel M, Miranda O, Diaz G, Diaz JE, Fleites YA, Pineiro CA, Castro CM, Koivogui L, Magassouba N, Diallo B, Ruibal P, Oestereich L, Wozniak DM, Ludtke A, Becker-Ziaja B, Capobianchi MR, Ippolito G, Carroll MW, Gunther S, Di Caro A, Munoz-Fontela C, Agrati C. Different features of Vdelta2 T and NK cells in fatal and non-fatal human Ebola infections. PLoS Negl Trop Dis. 2017;11(5):e0005645.

48. Alrubayyi A. NK cells in COVID-19: protectors or opponents? Nat Rev Immunol. 2020;20(9):520.

49. Witkowski M, Tizian C, Ferreira-Gomes M, Niemeyer D, Jones TC, Heinrich F, Frischbutter S, Angermair S, Hohnstein T, Mattiola I, Nawrath P, McEwen S, Zocche S, Viviano E, Heinz GA, Maurer M, Kolsch U, Chua RL, Aschman T, Meisel C, Radke J, Sawitzki B, Roehmel J, Allers K, Moos V, Schneider T, Hanitsch L, Mall MA, Conrad C, Radbruch H, Duerr CU, Trapani JA, Marcenaro E, Kallinich T, Corman VM, Kurth F, Sander LE, Drosten C, Treskatsch S, Durek P, Kruglov A, Radbruch A, Mashreghi MF, Diefenbach A. Untimely TGFbeta responses in COVID-19 limit antiviral functions of NK cells. Nature. 2021;600(7888):295-301.

50. Maucourant C, Filipovic I, Ponzetta A, Aleman S, Cornillet M, Hertwig L, Strunz B, Lentini A, Reinius B, Brownlie D, Cuapio A, Ask EH, Hull RM, Haroun-Izquierdo A, Schaffer M, Klingstrom J, Folkesson E, Buggert M, Sandberg JK, Eriksson LI, Rooyackers O, Ljunggren HG, Malmberg KJ, Michaelsson J, Marquardt N, Hammer Q, Stralin K, Bjorkstrom NK, Karolinska C-SG. Natural killer cell immunotypes related to COVID-19 disease severity. Sci Immunol. 2020;5(50).

51. Galan M, Vigon L, Fuertes D, Murciano-Anton MA, Casado-Fernandez G, Dominguez-Mateos S, Mateos E, Ramos-Martin F, Planelles V, Torres M, Rodriguez-Mora S, Lopez-Huertas MR, Coiras M. Persistent Overactive Cytotoxic Immune Response in a Spanish Cohort of Individuals With Long-COVID: Identification of Diagnostic Biomarkers. Front Immunol. 2022;13:848886.

52. Bi J. NK cell dysfunction in patients with COVID-19. Cell Mol Immunol. 2022;19(2):127-9.

53. Di Vito C, Calcaterra F, Coianiz N, Terzoli S, Voza A, Mikulak J, Della Bella S, Mavilio D. Natural Killer Cells in SARS-CoV-2 Infection: Pathophysiology and Therapeutic Implications. Front Immunol. 2022;13:888248.

54. Williams KJ, Qiu X, Fernando L, Jones SM, Alimonti JB. VSVDeltaG/EBOV GP-induced innate protection enhances natural killer cell activity to increase survival in a lethal mouse adapted Ebola virus infection. Viral Immunol. 2015;28(1):51-61.

55. Warfield KL, Perkins JG, Swenson DL, Deal EM, Bosio CM, Aman MJ, Yokoyama WM, Young HA, Bavari S. Role of natural killer cells in innate protection against lethal ebola virus infection. J Exp Med. 2004;200(2):169-79.

56. Wagstaffe HR, Clutterbuck EA, Bockstal V, Stoop JN, Luhn K, Douoguih M, Shukarev G, Snape MD, Pollard AJ, Riley EM, Goodier MR. Antibody-Dependent Natural Killer Cell Activation After Ebola Vaccination. J Infect Dis. 2021;223(7):1171-82.

57. Haveri A, Ekstrom N, Solastie A, Virta C, Osterlund P, Isosaari E, Nohynek H, Palmu AA, Melin M. Persistence of neutralizing antibodies a year after SARS-CoV-2 infection in humans. Eur J Immunol. 2021;51(12):3202-13.

58. Gussarow D, Bonifacius A, Cossmann A, Stankov MV, Mausberg P, Tischer-Zimmermann S, Godecke N, Kalinke U, Behrens GMN, Blasczyk R, Eiz-Vesper B. Long-Lasting Immunity Against SARS-CoV-2: Dream or Reality? Front Med (Lausanne). 2021;8:770381.

59. Petersen MS, Hansen CB, Kristiansen MF, Fjallsbak JP, Larsen S, Hansen JL, Jarlhelt I, Perez-Alos L, Steig BA, Christiansen DH, Moller LF, Strom M, Andorsdottir G, Gaini S, Weihe P, Garred P. SARS-CoV-2 Natural Antibody Response Persists for at Least 12 Months in a Nationwide Study From the Faroe Islands. Open Forum Infect Dis. 2021;8(8):ofab378.

60. Maciola AK, La Raja M, Pacenti M, Salata C, De Silvestro G, Rosato A, Pasqual G. Neutralizing Antibody Responses to SARS-CoV-2 in Recovered COVID-19 Patients Are Variable and Correlate With Disease Severity and Receptor-Binding Domain Recognition. Front Immunol. 2022;13:830710.

61. Gupta SL, Jaiswal RK. Neutralizing antibody: a savior in the Covid-19 disease. Mol Biol Rep. 2022;49(3):2465-74.

62. Cromer D, Steain M, Reynaldi A, Schlub TE, Wheatley AK, Juno JA, Kent SJ, Triccas JA, Khoury DS, Davenport MP. Neutralising antibody titres as predictors of protection against SARS-CoV-2 variants and the impact of boosting: a meta-analysis. Lancet Microbe. 2022;3(1):e52-e61.

63. Arunkumar G, Devadiga S, McElroy AK, Prabhu S, Sheik S, Abdulmajeed J, Robin S, Sushama A, Jayaram A, Nittur S, Shakir M, Kumar KGS, Radhakrishnan C, Sakeena K, Vasudevan J, Reena KJ, Sarita RL, Klena JD, Spiropoulou CF, Laserson KF, Nichol ST. Adaptive Immune Responses in Humans During Nipah Virus Acute and Convalescent Phases of Infection. Clin Infect Dis. 2019;69(10):1752-6.

64. Berhane Y, Weingartl HM, Lopez J, Neufeld J, Czub S, Embury-Hyatt C, Goolia M, Copps J, Czub M. Bacterial infections in pigs experimentally infected with Nipah virus. Transbound Emerg Dis. 2008;55(3-4):165-74.

65. Geisbert TW, Mire CE, Geisbert JB, Chan YP, Agans KN, Feldmann F, Fenton KA, Zhu Z, Dimitrov DS, Scott DP, Bossart KN, Feldmann H, Broder CC. Therapeutic treatment of Nipah virus infection in nonhuman primates with a neutralizing human monoclonal antibody. Sci Transl Med. 2014;6(242):242ra82.

66. Mire CE, Chan YP, Borisevich V, Cross RW, Yan L, Agans KN, Dang HV, Veesler D, Fenton KA, Geisbert TW, Broder CC. A Cross-Reactive Humanized Monoclonal Antibody Targeting Fusion Glycoprotein Function Protects Ferrets Against Lethal Nipah Virus and Hendra Virus Infection. J Infect Dis. 2020;221(Suppl 4):S471-S9.

67. Bossart KN, Zhu Z, Middleton D, Klippel J, Crameri G, Bingham J, McEachern JA, Green D, Hancock TJ, Chan YP, Hickey AC, Dimitrov DS, Wang LF, Broder CC. A neutralizing human monoclonal antibody protects against lethal disease in a new ferret model of acute nipah virus infection. PLoS Pathog. 2009;5(10):e1000642.

68. Guillaume V, Contamin H, Loth P, Grosjean I, Courbot MC, Deubel V, Buckland R, Wild TF. Antibody prophylaxis and therapy against Nipah virus infection in hamsters. J Virol. 2006;80(4):1972-8.

69. Guillaume V, Contamin H, Loth P, Georges-Courbot MC, Lefeuvre A, Marianneau P, Chua KB, Lam SK, Buckland R, Deubel V, Wild TF. Nipah virus: vaccination and passive protection studies in a hamster model. J Virol. 2004;78(2):834-40.

70. Dang HV, Cross RW, Borisevich V, Bornholdt ZA, West BR, Chan YP, Mire CE, Da Silva SC, Dimitrov AS, Yan L, Amaya M, Navaratnarajah CK, Zeitlin L, Geisbert TW, Broder CC, Veesler D. Broadly neutralizing antibody cocktails targeting Nipah virus and Hendra virus fusion glycoproteins. Nat Struct Mol Biol. 2021;28(5):426-34.

71. Gao Z, Li T, Han J, Feng S, Li L, Jiang Y, Xu Z, Hao P, Chen J, Hao J, Xu P, Tian M, Jin N, Huang W, Li C. Assessment of the immunogenicity and protection of a Nipah virus soluble G vaccine candidate in mice and pigs. Front Microbiol. 2022;13:1031523.

72. Zhu Z, Dimitrov AS, Bossart KN, Crameri G, Bishop KA, Choudhry V, Mungall BA, Feng YR, Choudhary A, Zhang MY, Feng Y, Wang LF, Xiao X, Eaton BT, Broder CC, Dimitrov DS. Potent neutralization of Hendra and Nipah viruses by human monoclonal antibodies. J Virol. 2006;80(2):891-9.

73. Longet S, Mellors J, Carroll MW, Tipton T. Ebolavirus: Comparison of Survivor Immunology and Animal Models in the Search for a Correlate of Protection. Front Immunol. 2020;11:599568.

74. Marzi A, Engelmann F, Feldmann F, Haberthur K, Shupert WL, Brining D, Scott DP, Geisbert TW, Kawaoka Y, Katze MG, Feldmann H, Messaoudi I. Antibodies are necessary for rVSV/ZEBOV-GP-mediated protection against lethal Ebola virus challenge in nonhuman primates. Proc Natl Acad Sci U S A. 2013;110(5):1893-8.

75. Rimoin AW, Lu K, Bramble MS, Steffen I, Doshi RH, Hoff NA, Mukadi P, Nicholson BP, Alfonso VH, Olinger G, Sinai C, Yamamoto LK, Ramirez CM, Okitolonda Wemakoy E, Kebela Illunga B, Pettitt J, Logue J, Bennett RS, Jahrling P, Heymann DL, Piot P, Muyembe-Tamfum JJ, Hensley LE, Simmons G. Ebola Virus Neutralizing Antibodies Detectable in Survivors of theYambuku, Zaire Outbreak 40 Years after Infection. J Infect Dis. 2018;217(2):223-31.

76. Saphire EO, Schendel SL, Gunn BM, Milligan JC, Alter G. Antibody-mediated protection against Ebola virus. Nat Immunol. 2018;19(11):1169-78.

77. Gunn BM, Yu WH, Karim MM, Brannan JM, Herbert AS, Wec AZ, Halfmann PJ, Fusco ML, Schendel SL, Gangavarapu K, Krause T, Qiu X, He S, Das J, Suscovich TJ, Lai J, Chandran K, Zeitlin L, Crowe JE, Jr., Lauffenburger D, Kawaoka Y, Kobinger GP, Andersen KG, Dye JM, Saphire EO, Alter G. A Role for Fc Function in Therapeutic Monoclonal Antibody-Mediated Protection against Ebola Virus. Cell Host Microbe. 2018;24(2):221-33 e5.

78. Maruyama T, Rodriguez LL, Jahrling PB, Sanchez A, Khan AS, Nichol ST, Peters CJ, Parren PW, Burton DR. Ebola virus can be effectively neutralized by antibody produced in natural human infection. J Virol. 1999;73(7):6024-30.

79. Gunn BM, Roy V, Karim MM, Hartnett JN, Suscovich TJ, Goba A, Momoh M, Sandi JD, Kanneh L, Andersen KG, Shaffer JG, Schieffelin JS, Garry RF, Grant DS, Alter G. Survivors of Ebola Virus Disease Develop Polyfunctional Antibody Responses. J Infect Dis. 2020;221(1):156-61.

80. Castillo-Olivares J, Wells DA, Ferrari M, Chan ACY, Smith P, Nadesalingam A, Paloniemi M, Carnell GW, Ohlendorf L, Cantoni D, Mayora-Neto M, Palmer P, Tonks P, Temperton NJ, Peterhoff D, Neckermann P, Wagner R, Doffinger R, Kempster S, Otter AD, Semper A, Brooks T, Albecka A, James LC, Page M, Schwaeble W, Baxendale H, Heeney JL. Analysis of Serological Biomarkers of SARS-CoV-2 Infection in Convalescent Samples From Severe, Moderate and Mild COVID-19 Cases. Front Immunol. 2021;12:748291.

81. Alfego D, Sullivan A, Poirier B, Williams J, Adcock D, Letovsky S. A population-based analysis of the longevity of SARS-CoV-2 antibody seropositivity in the United States. EClinicalMedicine. 2021;36:100902.

82. Chan YP, Lu M, Dutta S, Yan L, Barr J, Flora M, Feng YR, Xu K, Nikolov DB, Wang LF, Skiniotis G, Broder CC. Biochemical, conformational, and immunogenic analysis of soluble trimeric forms of henipavirus fusion glycoproteins. J Virol. 2012;86(21):11457-71.

83. DeBuysscher BL, Scott D, Marzi A, Prescott J, Feldmann H. Single-dose live-attenuated Nipah virus vaccines confer complete protection by eliciting antibodies directed against surface glycoproteins. Vaccine. 2014;32(22):2637-44.

84. Bornholdt ZA, Turner HL, Murin CD, Li W, Sok D, Souders CA, Piper AE, Goff A, Shamblin JD, Wollen SE, Sprague TR, Fusco ML, Pommert KB, Cavacini LA, Smith HL, Klempner M, Reimann KA, Krauland E, Gerngross TU, Wittrup KD, Saphire EO, Burton DR, Glass PJ, Ward AB, Walker LM. Isolation of potent neutralizing antibodies from a survivor of the 2014 Ebola virus outbreak. Science. 2016;351(6277):1078-83.

85. Albecka A, Clift D, Vaysburd M, Rhinesmith T, Caddy SL, Favara DM, Baxendale HE, James LC. A functional assay for serum detection of antibodies against SARS-CoV-2 nucleoprotein. EMBO J. 2021;40(17):e108588.

86. Dufloo J, Grzelak L, Staropoli I, Madec Y, Tondeur L, Anna F, Pelleau S, Wiedemann A, Planchais C, Buchrieser J, Robinot R, Ungeheuer MN, Mouquet H, Charneau P, White M, Levy Y, Hoen B, Fontanet A, Schwartz O, Bruel T. Asymptomatic and symptomatic SARS-CoV-2 infections elicit polyfunctional antibodies. Cell Rep Med. 2021;2(5):100275.

87. Yu Y, Wang M, Zhang X, Li S, Lu Q, Zeng H, Hou H, Li H, Zhang M, Jiang F, Wu J, Ding R, Zhou Z, Liu M, Si W, Zhu T, Li H, Ma J, Gu Y, She G, Li X, Zhang Y, Peng K, Huang W, Liu W, Wang Y. Antibody-dependent cellular cytotoxicity response to SARS-CoV-2 in COVID-19 patients. Signal Transduct Target Ther. 2021;6(1):346.

88. Bates TA, Lu P, Kang YJ, Schoen D, Thornton M, McBride SK, Park C, Kim D, Messer WB, Curlin ME, Tafesse FG, Lu LL. BNT162b2-induced neutralizing and non-neutralizing antibody functions against SARS-CoV-2 diminish with age. Cell Rep. 2022;41(4):111544.

89. Keshwara R, Shiels T, Postnikova E, Kurup D, Wirblich C, Johnson RF, Schnell MJ. Rabies-based vaccine induces potent immune responses against Nipah virus. NPJ Vaccines. 2019;4:15.

90. Kuzmina NA, Younan P, Gilchuk P, Santos RI, Flyak AI, Ilinykh PA, Huang K, Lubaki NM, Ramanathan P, Crowe JE, Jr., Bukreyev A. Antibody-Dependent Enhancement of Ebola Virus Infection by Human Antibodies Isolated from Survivors. Cell Rep. 2018;24(7):1802-15 e5.

91. Goldblatt D, Alter G, Crotty S, Plotkin SA. Correlates of protection against SARS-CoV-2 infection and COVID-19 disease. Immunol Rev. 2022;310(1):6-26.

92. Iles JK, Zmuidinaite R, Sadee C, Gardiner A, Lacey J, Harding S, Wallis G, Patel R, Roblett D, Heeney J, Baxendale H, Iles RK. Determination of IgG1 and IgG3 SARS-CoV-2 Spike Protein and Nucleocapsid Binding-Who Is Binding Who and Why? Int J Mol Sci. 2022;23(11).

93. Zhu Z, Bossart KN, Bishop KA, Crameri G, Dimitrov AS, McEachern JA, Feng Y, Middleton D, Wang LF, Broder CC, Dimitrov DS. Exceptionally potent cross-reactive neutralization of Nipah and Hendra viruses by a human monoclonal antibody. J Infect Dis. 2008;197(6):846-53.

94. Davis CW, Jackson KJL, McElroy AK, Halfmann P, Huang J, Chennareddy C, Piper AE, Leung Y, Albarino CG, Crozier I, Ellebedy AH, Sidney J, Sette A, Yu T, Nielsen SCA, Goff AJ, Spiropoulou CF, Saphire EO, Cavet G, Kawaoka Y, Mehta AK, Glass PJ, Boyd SD, Ahmed R. Longitudinal Analysis of the Human B Cell Response to Ebola Virus Infection. Cell. 2019;177(6):1566-82 e17.

95. Havervall S, Marking U, Svensson J, Greilert-Norin N, Bacchus P, Nilsson P, Hober S, Gordon M, Blom K, Klingstrom J, Aberg M, Smed-Sorensen A, Thalin C. Anti-Spike Mucosal IgA Protection against SARS-CoV-2 Omicron Infection. N Engl J Med. 2022;387(14):1333-6.

96. Fraser R, Orta-Resendiz A, Mazein A, Dockrell DH. Upper respiratory tract mucosal immunity for SARS-CoV-2 vaccines. Trends Mol Med. 2023;29(4):255-67.

97. Tang J, Zeng C, Cox TM, Li C, Son YM, Cheon IS, Wu Y, Behl S, Taylor JJ, Chakaraborty R, Johnson AJ, Shiavo DN, Utz JP, Reisenauer JS, Midthun DE, Mullon JJ, Edell ES, Alameh MG, Borish L, Teague WG, Kaplan MH, Weissman D, Kern R, Hu H, Vassallo R, Liu SL, Sun J. Respiratory mucosal immunity against SARS-CoV-2 after mRNA vaccination. Sci Immunol. 2022;7(76):eadd4853.

98. Lapuente D, Fuchs J, Willar J, Vieira Antao A, Eberlein V, Uhlig N, Issmail L, Schmidt A, Oltmanns F, Peter AS, Mueller-Schmucker S, Irrgang P, Fraedrich K, Cara A, Hoffmann M, Pohlmann S, Ensser A, Pertl C, Willert T, Thirion C, Grunwald T, Uberla K, Tenbusch M. Protective mucosal immunity against SARS-CoV-2 after heterologous systemic prime-mucosal boost immunization. Nat Commun. 2021;12(1):6871.

99. Houston S. SARS-CoV-2 mucosal vaccine. Nat Immunol. 2023;24(1):1.

100. McEachern JA, Bingham J, Crameri G, Green DJ, Hancock TJ, Middleton D, Feng YR, Broder CC, Wang LF, Bossart KN. A recombinant subunit vaccine formulation protects against lethal Nipah virus challenge in cats. Vaccine. 2008;26(31):3842-52.

101. Khurana S, Fuentes S, Coyle EM, Ravichandran S, Davey RT, Jr., Beigel JH. Human antibody repertoire after VSV-Ebola vaccination identifies novel targets and virus-neutralizing IgM antibodies. Nat Med. 2016;22(12):1439-47.

102. Notarbartolo S, Ranzani V, Bandera A, Gruarin P, Bevilacqua V, Putignano AR, Gobbini A, Galeota E, Manara C, Bombaci M, Pesce E, Zagato E, Favalli A, Sarnicola ML, Curti S, Crosti M, Martinovic M, Fabbris T, Marini F, Donnici L, Lorenzo M, Mancino M, Ungaro R, Lombardi A, Mangioni D, Muscatello A, Aliberti S, Blasi F, De Feo T, Prati D, Manganaro L, Granucci F, Lanzavecchia A, De Francesco R, Gori A, Grifantini R, Abrignani S. Integrated longitudinal immunophenotypic, transcriptional and repertoire analyses delineate immune responses in COVID-19 patients. Sci Immunol. 2021;6(62).

103. Su Y, Chen D, Yuan D, Lausted C, Choi J, Dai CL, Voillet V, Duvvuri VR, Scherler K, Troisch P. Multi-omics resolves a sharp disease-state shift between mild and moderate COVID-19. Cell. 2020;183(6):1479-95. e20.

104. Moss P. The T cell immune response against SARS-CoV-2. Nat Immunol. 2022;23(2):186-93.

105. Bruno L, Nappo MA, Ferrari L, Di Lecce R, Guarnieri C, Cantoni AM, Corradi A. Nipah Virus Disease: Epidemiological, Clinical, Diagnostic and Legislative Aspects of This Unpredictable Emerging Zoonosis. Animals (Basel). 2022;13(1).

106. Tipton TRW, Hall Y, Bore JA, White A, Sibley LS, Sarfas C, Yuki Y, Martin M, Longet S, Mellors J, Ewer K, Gunther S, Carrington M, Konde MK, Carroll MW. Characterisation of the T-cell response to Ebola virus glycoprotein amongst survivors of the 2013-16 West Africa epidemic. Nat Commun. 2021;12(1):1153.

107. Thom R, Tipton T, Strecker T, Hall Y, Akoi Bore J, Maes P, Raymond Koundouno F, Fehling SK, Krahling V, Steeds K, Varghese A, Bailey G, Matheson M, Kouyate S, Cone M, Moussa Keita B, Kouyate S, Richard Ablam A, Laenen L, Vergote V, Guiver M, Timothy J, Atkinson B, Ottowell L, Richards KS, Bosworth A, Longet S, Mellors J, Pannetier D, Duraffour S, Munoz-Fontela C, Sow O, Koivogui L, Newman E, Becker S, Sprecher A, Raoul H, Hiscox J, Henao-Restrepo AM, Sakoba K, Magassouba N, Gunther S, Kader Konde M, Carroll MW. Longitudinal antibody and T cell responses in Ebola virus disease survivors and contacts: an observational cohort study. Lancet Infect Dis. 2021;21(4):507-16.

108. Wang Z, Yang X, Zhong J, Zhou Y, Tang Z, Zhou H, He J, Mei X, Tang Y, Lin B, Chen Z, McCluskey J, Yang J, Corbett AJ, Ran P. Exposure to SARS-CoV-2 generates T-cell memory in the absence of a detectable viral infection. Nat Commun. 2021;12(1):1724.

109. Ssemaganda A, Nguyen HM, Nuhu F, Jahan N, Card CM, Kiazyk S, Severini G, Keynan Y, Su RC, Ji H, Abrenica B, McLaren PJ, Ball TB, Bullard J, Van Caeseele P, Stein D, McKinnon LR. Expansion of cytotoxic tissue-resident CD8(+) T cells and CCR6(+)CD161(+) CD4(+) T cells in the nasal mucosa following mRNA COVID-19 vaccination. Nat Commun. 2022;13(1):3357.

110. Jain S, Khaiboullina SF, Baranwal M. Immunological Perspective for Ebola Virus Infection and Various Treatment Measures Taken to Fight the Disease. Pathogens. 2020;9(10).

111. Speranza E, Ruibal P, Port JR, Feng F, Burkhardt L, Grundhoff A, Gunther S, Oestereich L, Hiscox JA, Connor JH, Munoz-Fontela C. T-Cell Receptor Diversity and the Control of T-Cell Homeostasis Mark Ebola Virus Disease Survival in Humans. J Infect Dis. 2018;218(suppl_5):S508-S18.
